# US State Restrictions and Excess COVID-19 Pandemic Deaths

**DOI:** 10.1001/jamahealthforum.2024.2006

**Published:** 2024-07-26

**Authors:** Christopher J. Ruhm

**Affiliations:** 1 Frank Batten School of Leadership and Public Policy, University of Virginia, Charlottesville; 2 National Bureau of Economic Research, Cambridge, Massachusetts

## Abstract

**Importance:**

Despite considerable prior research, it remains unclear whether and by how much state COVID-19−related restrictions affected the number of pandemic deaths in the US.

**Objective:**

To determine how state restrictions were associated with excess COVID-19 deaths over a 2-year analysis period.

**Design, Setting, and Participants:**

This was a cross-sectional study using state-level mortality and population data from the US Centers for Disease Control and Prevention for 2020 to 2022 compared with baseline data for 2017 to 2019. Data included the total US population, with separate estimates for younger than 45 years, 45 to 64 years, 65 to 84 years, and 85 years or older used to construct age-standardized measures. Age-standardized excess mortality rates and ratios for July 2020 to June 2022 were calculated and compared with prepandemic baseline rates. Excess death rates and ratios were then regressed on single or multiple restrictions, while controlling for excess death rates or ratios, from March 2020 to June 2020. Estimated values of the dependent variables were calculated for packages of weak vs strong state restrictions. Behavioral changes were investigated as a potential mechanism for the overall effects. Data analyses were performed from October 1, 2023, to June 13, 2024.

**Exposures:**

Age and cause of death.

**Main Outcomes:**

Excess deaths, age-standardized excess death rates per 100 000, and excess death ratios.

**Results:**

Mask requirements and vaccine mandates were negatively associated with excess deaths, prohibitions on vaccine or mask mandates were positively associated with death rates, and activity limitations were mostly not associated with death rates. If all states had imposed restrictions similar to those used in the 10 most restrictive states, excess deaths would have been an estimated 10% to 21% lower than the 1.18 million that actually occurred during the 2-year analysis period; conversely, the estimates suggest counterfactual increases of 13% to 17% if all states had restrictions similar to those in the 10 least-restrictive states. The estimated strong vs weak state restriction difference was 271 000 to 447 000 deaths, with behavior changes associated with 49% to 79% of the overall disparity.

**Conclusions and Relevance:**

This cross-sectional study indicates that stringent COVID-19 restrictions, as a group, were associated with substantial decreases in pandemic mortality, with behavior changes plausibly serving as an important explanatory mechanism. These findings do not support the views that COVID-19 restrictions were ineffective. However, not all restrictions were equally effective; some, such as school closings, likely provided minimal benefit while imposing substantial cost.

## Key Points

**Question:**

How did state restrictions affect the number of excess COVID-19 pandemic deaths?

**Findings:**

This cross-sectional analysis including all 50 US states plus the District of Columbia found that if all states had imposed COVID-19 restrictions similar to those used in the 10 most (least) restrictive states, excess deaths would have been an estimated 10% to 21% lower (13%-17% higher) than the 1.18 million that actually occurred during the 2-year period analyzed. Behavior changes were associated with 49% to 79% of this overall difference.

**Meaning:**

These findings indicate that collectively, stringent COVID-19 restrictions were associated with substantial decreases in excess deaths during the pandemic.

## Teaser

This cross-sectional study uses state-level mortality and population data for 2020 to 2022 to determine whether the strength of state COVID-19 restrictions were associated with excess pandemic deaths in the US.

## Introduction

US states swiftly responded to the emergence of COVID-19 by implementing a variety of policies including initial emergency declarations, diverse forms of shutdowns and, later, mask and vaccine mandates. The initial actions were almost universal,^1^ but opposition to these measures quickly developed.^2,3^ This dissent spilled into the political arena where Republican governors and legislatures in multiple states limited the authority of public health officials and banned mask and vaccine mandates.^4,5^

Numerous studies have evaluated the impact of policies designed to mitigate the COVID-19 pandemic.^6,7,8^ Early analyses suggested that strong social distancing measures curtailed the initial spread of COVID-19,^9,10^ with evidence of interactive effects between public and private mitigation efforts.^11^ However, the specific modeling assumptions and design criteria of some of these studies have been questioned,^3,12,13^ and these analyses generally covered only the initial stages of the pandemic.

Obtaining causal estimates is complicated because of unobserved confounding factors and possible reverse causation, particularly given that the pandemic occurred over a relatively restricted time period and its dynamics are challenging to model. These general issues are exacerbated by preexisting geographic differences in population and employment characteristics, behaviors, and policy environments that affect mortality.^5,14,15^ An additional challenge is that initial COVID-19 exposure was concentrated in a small number of locations: primarily New York state, its surrounding states, and Massachusetts. These initial infection rates were probably largely idiosyncratic, reflecting factors such as having high population densities, being travel destinations, and making extensive use of public transportation.^16,17,18^ During this early period, policy variation across states was limited^1^ and the lethality of COVID-19 was especially high^19^ because medical professionals had little experience with and few tools available to treat severe COVID-19 infections. Consequently, studies containing data for the early pandemic period, such as those examining the overall performance of states^20^ or groups of policy responses,^21^ faced particular challenges. Reverse causation also may have been problematic given that states with the worst initial conditions were likely to have implemented the most restrictive policies.^22^ Moreover, restrictions were often introduced as packages, which made it difficult to disentangle specific policy effects.

This analysis focuses on state policies aimed at limiting COVID-19−related deaths during the pandemic. The primary investigation covers the 2-year period July 2020 to June 2022. “Activity limitations” refers to mandated closures of restaurants, bars, and schools; stay-at-home orders; and restrictions on leisure time activities and public gatherings. Other limitations that were examined included general mask requirements and mask and vaccine mandates, as well as prohibitions on these measures. Strategies used to mitigate the estimation challenges described include (1) using 2 methods of accounting for state differences in age-standardized prepandemic death rates to provide likely upper and lower bounds on the restriction effects and (2) beginning the analysis approximately 4 months after the first COVID-19 deaths in the US to mitigate the largely idiosyncratic variation at the start of the pandemic. Of particular interest is the overall impact of packages of “weak” vs “strong” COVID-19 restrictions rather than individual policy effects because policy responses were generally strongly correlated. Lastly, the association of behavioral changes with estimated restriction effects and excess mortality is examined.

## Methods

This study followed the Strengthening the Reporting of Observational Studies in Epidemiology (STROBE) reporting guideline for cross-sectional studies.^23^ Because all data were obtained from public sources, this study did not constitute human participant research and did not require institutional review board review or exemption according to the US Department of Health and Human Services (45 CFR §46).

### Outcomes

Death rates per 100 000 were computed using population and mortality data obtained from CDC Wonder,^24,25^ age-standardized throughout based on 2020 national population shares for younger than 45 years, 45 to 64 years, 65 to 84 years, and 85 years or older. Pandemic deaths could be influenced by a variety of factors, such as differences in educational attainment, the industrial composition of employment, and the general policy environment. Although some of these could potentially be directly controlled for, others would likely not be, resulting in omitted variables biases. To address this, excess death rates, which have been widely used in the literature^26,27,28,29,30,31,32^ and excess death ratios were constructed.

Excess death rates were computed as actual death rates divided by baseline death rates during July 2017 to June 2019, a corresponding 2-year period before the COVID-19 pandemic. These accounted for determinants of cross-state differences in levels of baseline mortality, to the extent that they persisted during the pandemic. However, because death rates rose with the emergence of COVID-19, such adjustments in levels could be insufficient because equal percentage changes would raise rates by larger absolute amounts in states with higher rather than lower prepandemic mortality rates. If so, because states with lower baseline death rates adopted more restrictive policies, using excess death rates as the dependent variable might overstate the benefits of restrictions.

An alternative was to analyze excess death ratios computed as actual deaths divided by death baseline rates. These allowed for proportional adjustments in pandemic effects but were likely to underestimate the effectiveness of restrictions when the determinants of baseline and pandemic death rates differed. For example, persons living in nursing homes, areas with high population density, or making extensive use of public transportation may have been more vulnerable to COVID-19,^16,17,33^ while these factors were less closely associated with prepandemic mortality. In combination, analysis of excess death rates and ratios provided likely upper and lower bounds on the restriction effects.

### Restrictions

Data on 7 state activity limitations were obtained from the Institute for Health Metrics and Evaluation (IHME),^34^ including stay-at-home orders; restaurant, bar, leisure activity, primary school, and higher education closures; and restrictions on public gatherings. The percentage of days during which the limitations were in effect from July 2020 to June 2022 was calculated, and primary analysis focused on the population-weighted average of the 7 measures. Alternative sources of activity limitations were examined and shown to have similar patterns to those analyzed in this study.

The percentage of the analysis period during which all states required individuals to wear masks when outside of the home was calculated using information from Ballotpedia^35^; for the District of Columbia, data were from the IHME.^34^ Kaiser Family Foundation data^36^ were used to construct variables indicating whether state government or school employees were required to be vaccinated as of February 2022, whether there were school mask requirements, and whether there were prohibitions on government vaccine or school mask mandates.

### Behaviors

Three behaviors were examined. CDC vaccination data^37^ were used to calculate the percentage of the population completing a primary COVID-19 vaccination series. The percentage of days individuals stated that they always wore a mask outside their home (mask use) was obtained from IHME.^34^ Reductions in time outside the home compared with the prepandemic period (mobility reductions) came from Opportunity Insights.^38^

### Statistical Analysis

Descriptive analysis was conducted to evaluate patterns of activity limitations, mask requirements, behaviors, excess death rates, and ratios from March 2020 to December 2022. This analysis demonstrated the concentration of initial COVID-19 pandemic deaths in a few areas, despite the almost universal imposition of activity limitations, motivating use of the July 2020 to June 2022 analysis period. A composite restriction score was constructed as the sum of the standardized (mean [SD], 0 [1]) activity limitations score, and standardized mask requirements plus dummy variables for state and school vaccination and school mask requirements and minus the dummy variable values for vaccination and mask prohibitions.

Age-standardized excess death rates or ratios during the 2-year period, July 2020 to June 2022 (1 observation per state), were next regressed on single or multiple restrictions, controlling for excess death rates or ratios during the preanalysis period (March 2020-June 2020) in case later outcomes were influenced by early COVID-19 exposure, although preliminary analysis indicated this was only a minor issue. Observations were weighted by 2020 state populations, and confidence intervals were based on robust standard errors.

Given the collinearity between policies, the single restriction estimates likely provided upper bounds on magnitudes of the restriction effects. Therefore, particular emphasis was placed on results for packages of weak and strong restrictions, where the former (latter) were calculated using population-weighted averages for the 10 states with the lowest (highest) overall restriction scores. Specifically, weak restrictions referred to activity limitation scores and mask requirements of 0.80 and 1.41 SDs less than the national average; no vaccine or mask mandates; and vaccine and school mask prohibition values of 0.79 and 0.75, respectively. Strong restrictions referred to activity limitation scores and mask requirements of 1.07 and 1.26 SDs, respectively, greater than the national average; universal school and state vaccination mandates and school mask requirements; and no vaccine or mask mandate prohibitions (eTable 1 in Supplement 1).

The association between behavioral changes and restriction effects was also examined by regressing (1) the 3 behaviors on restrictions and (2) excess death rates or ratios on the estimated difference in behaviors associated with weak vs strong restrictions, with these then compared with the overall restriction effect. Additional details on the methods are provided in Supplement 1.

Statistical tests were 2-tailed and *P* <.05 were considered statistically significant. Data analyses were performed from October 1, 2023, to June 13, 2024, using Stata, release 18.0 (StataCorp LLC).

## Results

### Descriptive Patterns

**Figure 1.**
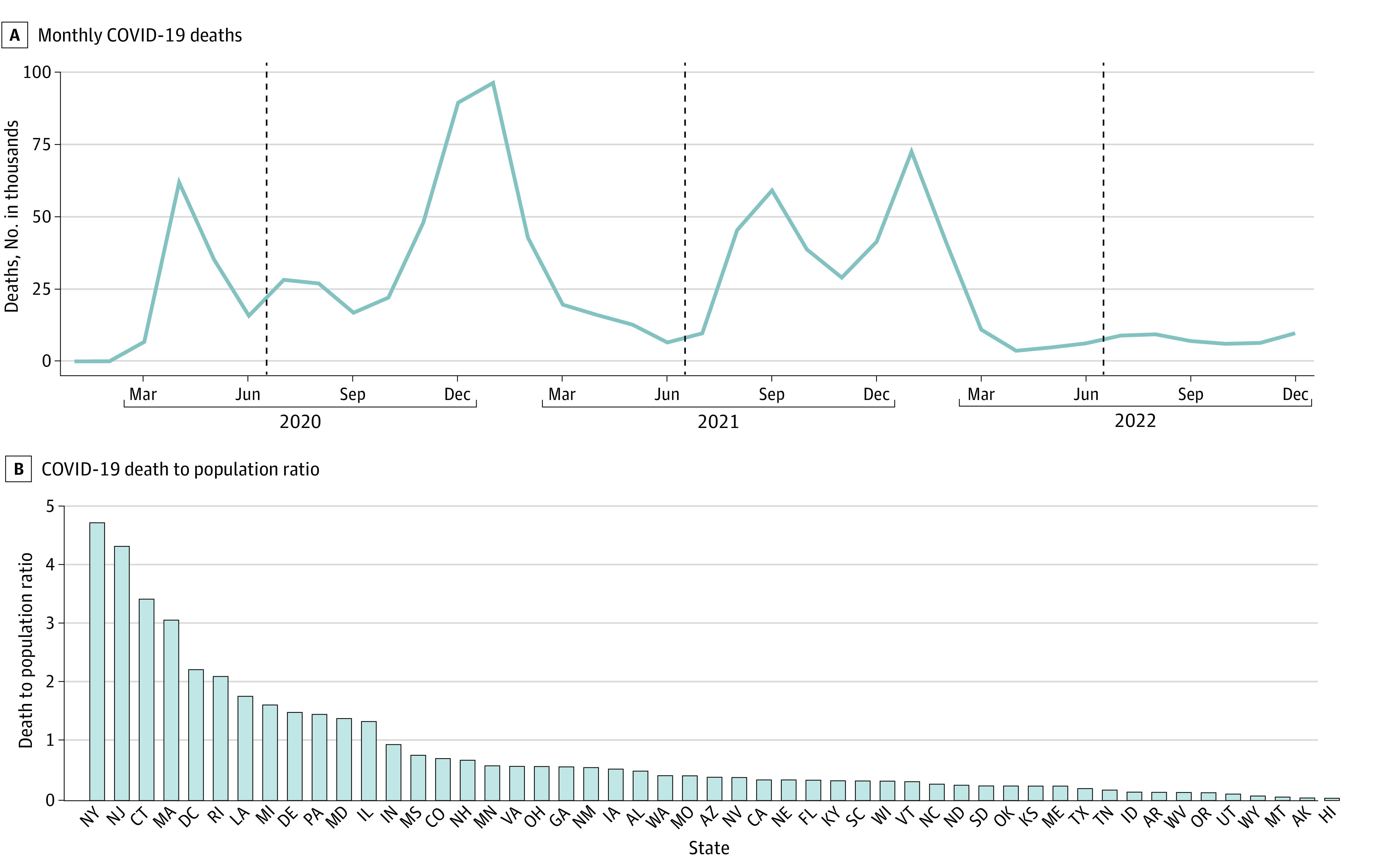
Monthly COVID-19 Deaths and Early Pandemic COVID-19 State Death-Population Ratio Monthly counts of deaths with COVID-19 as an underlying cause. Dotted vertical lines show analysis for year 1 (July 2020-June 2021) and year 2 (July 2021-June 2022) (A). National share of COVID-19 deaths occurring in the state from March 2020 to May 2020 divided by the state share of the national population (B).

**Figure 2.**
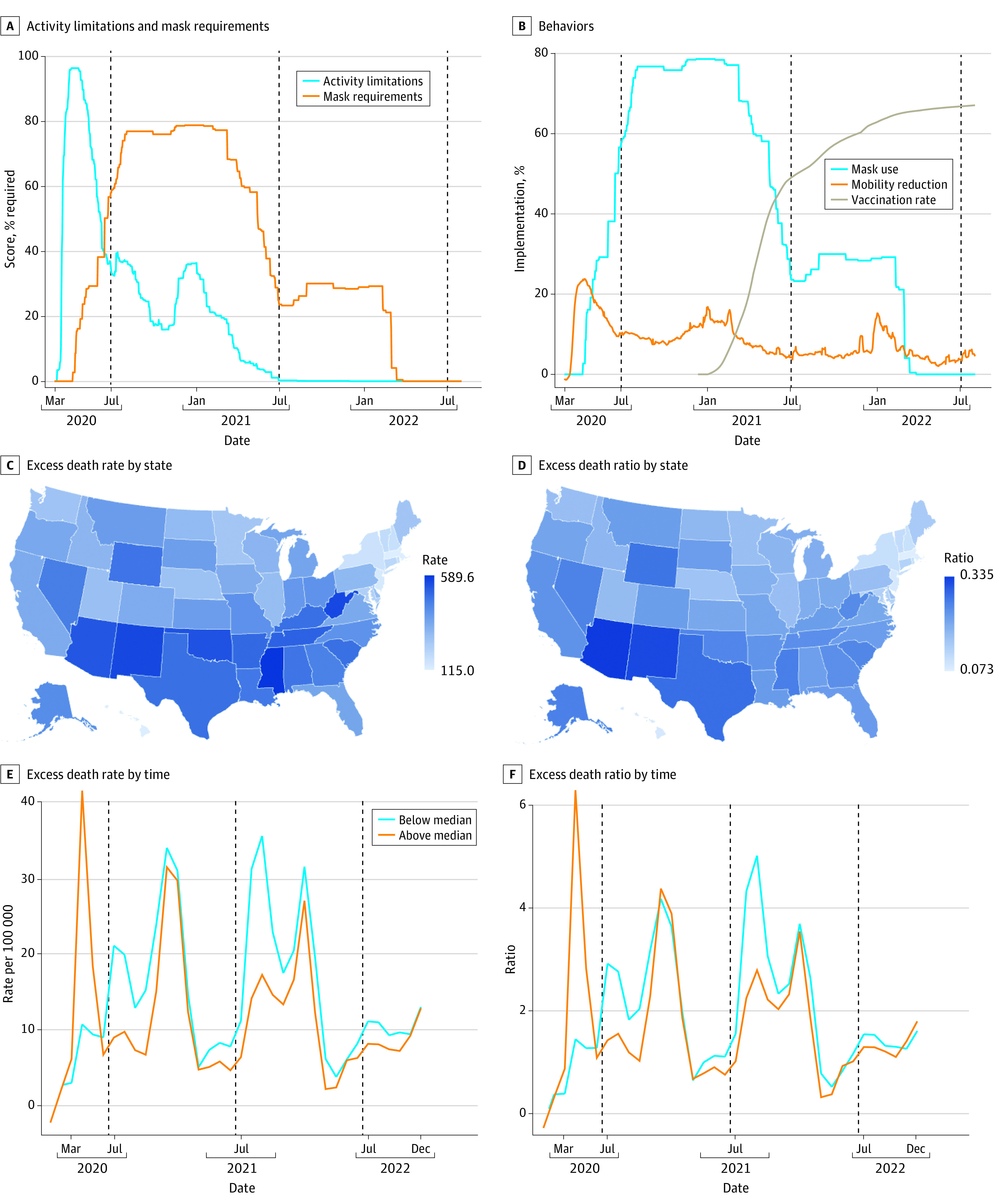
State Restrictions, Behaviors, and Age-Standardized Excess COVID-19 Death Rates and Ratios A, Percentage of individuals subject to 7 activity restrictions and required to wear a mask outside the home. B, Daily percentage of adults stating they always wear a mask when leaving home and reductions in time away from home compared to before the pandemic, and the percentage having received 1 dose of the Johnson & Johnson vaccine or 2 doses of the Pfizer or Moderna vaccines. C and D, Age-standardized death rates and ratios for July 2020 to June 2022. Age-standardized rates account for population shares of younger than 45 years, 45 to 64 years, 65 to 84 years, and 85 years or older. E and F, Age-standardized monthly excess death rates and ratios for subsamples with greater than/less than median restriction scores, calculated as the sum of population-weighted mean values of standardized activity limitation scores, mask requirements, and mask and vaccination mandates minus state prohibitions on vaccination mandates and mask requirements in schools. Dotted lines separate July 2020 to June 2021 from July 2021 to June 2022.

In the US, COVID-19 deaths emerged in March 2020. After subsiding, they became more numerous and geographically distributed from October 2020 through March 2021, with additional smaller peaks near the end of 2021 and beginning of 2022 when the delta and omicron variants emerged (Figure 1A). The pattern of excess death rates was nearly identical (eFigure 1 in Supplement 1). The initial surge, from March 2020 to May 2020, was highly concentrated in 4 states (Connecticut, Massachusetts, New Jersey, and New York) containing 12% of the nation’s population but accounting for half of the COVID-19 fatalities (Figure 1B; eTable 2 in Supplement 1). During this period, there was little variation in COVID-19 restrictions. For instance, all states had declared a state of emergency by March 15, 2020,^39^ the composite activity limitations score averaged 96.2 (of a maximum of 100) by April 7, 2020, and there were strong simultaneous behavioral responses, including large mobility reductions and increases in mask use (Figure 2A and B).

Considerable policy variation emerged in the second half of 2020, with states reducing or eliminating activity limitations and, somewhat later, mask requirements. Mobility reductions also declined rapidly during this period, as did mask use after the start of 2021. Vaccinations first became available in December 2020 and quickly became widespread, but with considerable geographic heterogeneity. Activity limitations had been essentially phased out by June 2021 and mask requirements by March 2022, at which point 23 and 11 states had instituted vaccination mandates for state and school employees, respectively, and 15 states had mandated masks in schools. Conversely, 13 states had prohibited vaccination mandates and 7 had outlawed school mask requirements (eTable 3 in Supplement 1).

With the exception of Hawaii, the states with the lowest excess death rates and ratios were in the Northeast region, while a mix of Southern and Western states had the highest rates and ratios (Figure 2C and D). At the extreme, the excess death rate in Massachusetts was less than one-fifth that of Mississippi (115 vs 590 per 100 000). The poor performance of Southern states was modestly mitigated when examining excess death ratios because of their high prepandemic death rates, but the population-weighted correlation between rates and ratios was high (0.91) and the rank ordering of states was largely unchanged. Relative performance was also similar when examining COVID-19 rather than all-cause excess death rates (eTable 4 in Supplement 1).

Figure 2E and F show monthly excess death rates and ratios for states with composite restriction scores greater than or less than the national median. Stronger restrictions were associated with lower monthly excess death rates and ratios in virtually every month from July 2020 to June 2022, with the largest gaps during the second half of 2020, when the virus first became widespread, and in September through December 2021 when the delta variant became dominant. However, strong restriction states had much higher excess death rates and ratios in the preanalysis period (March 2020 to June 2020). This early COVID-19 exposure may have lowered subsequent mortality risk; however, additional analysis revealed that these effects were minimal (eFigure 2 in Supplement 1).

### Single Restrictions and Behaviors

**Figure 3.**
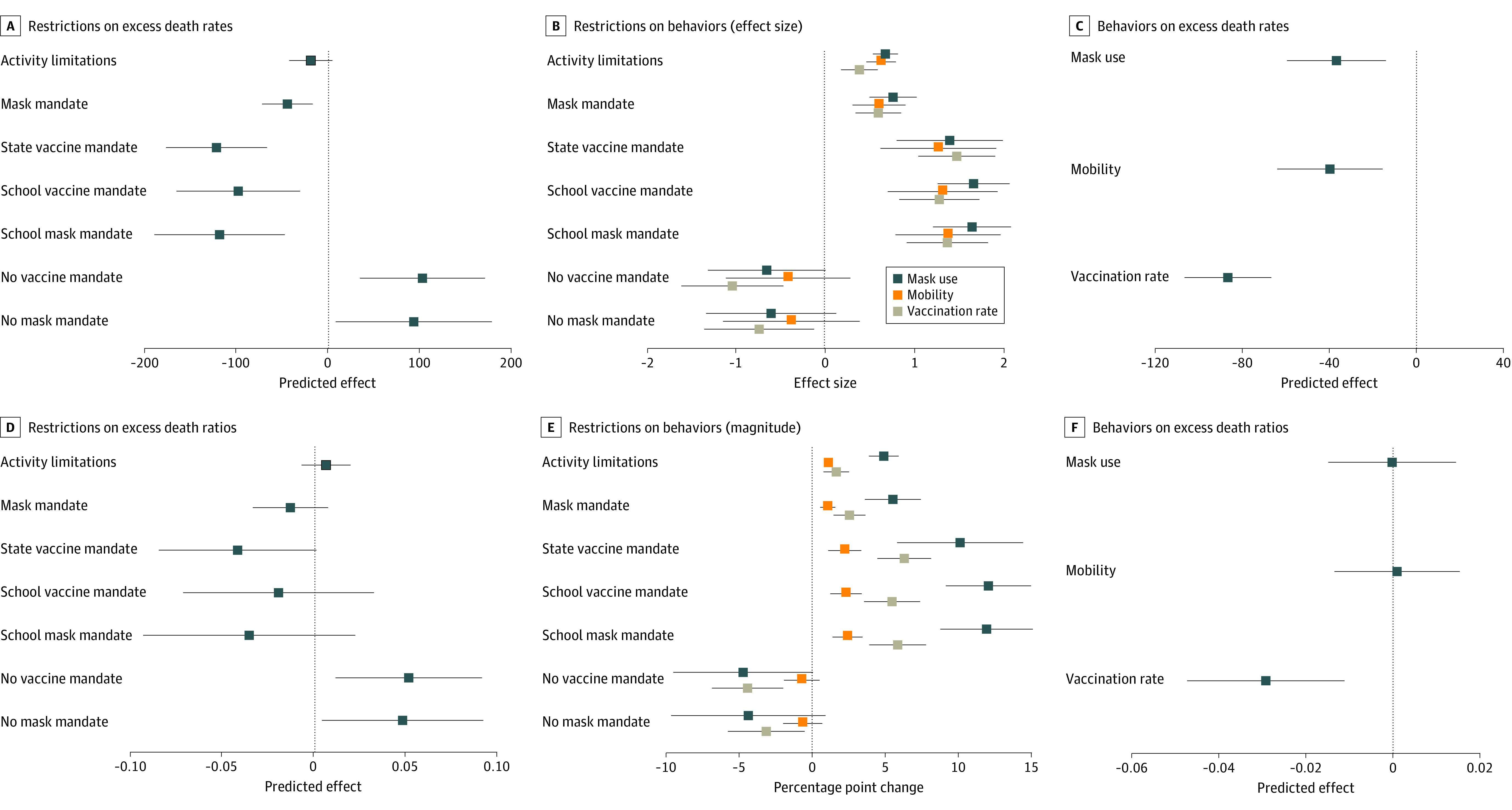
Estimated Effects of Individual COVID-19 Restrictions and Behaviors Coefficient estimates from regressing age-standardized state excess death rates and ratios from July 2020 to June 2022 on the single specified restriction (N = 51). Continuous variables are standardized so that the coefficients indicate the estimated effect of a 1-SD change. For dummy variables, the coefficient indicates the estimated effect of its value going from 0 to 1 (A, B). Dependent variables are values of the 3 behaviors (mask use, mobility reductions, and full vaccination rates) normalized to have a SD of 1 (C) with the same estimates for raw (not normalized) values (D). Estimated effects of a 1-SD change in the behaviors on age-standardized state excess death rates and ratios (E, F). Observations are weighted by 2020 state populations and error bars show 95% CIs based on robust standard errors. The analysis controlled for excess death rates or ratios from March 2020 to June 2020.

In regression models controlling for a single restriction variable, mask requirements and vaccine or school mask mandates were associated with reductions in estimated excess death rates and ratios; prohibitions on mask or vaccine mandates were positively associated with them; whereas activity limitations were largely unrelated (Figure 3A and B; eTables 5 and 6 in Supplement 1). Restrictions were associated with greater use of the 3 preventive behaviors (Figure 3C and D; eTable 5 in Supplement 1). These, in turn, were negatively correlated with excess death rates and, for vaccinations, with lower excess death ratios (eTable 7 in Supplement 1).

### Weak vs Strong Restrictions

**Figure 4.**
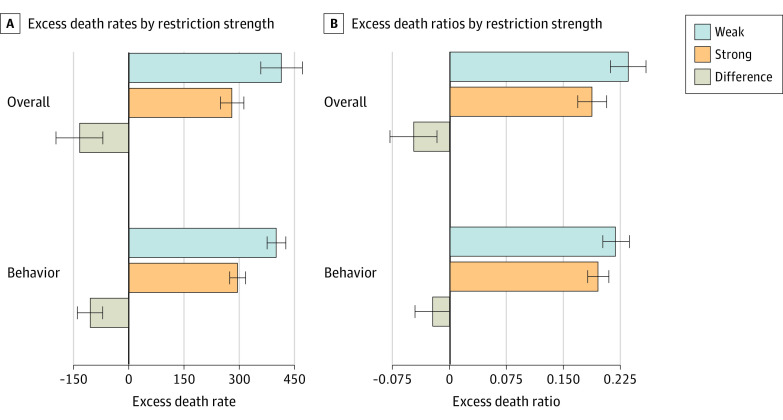
Estimated Effect of Weak and Strong COVID-19 Restrictions on Age-Standardized Excess Death Rates or Ratios and Role of Behavioral Responses The overall figure shows the estimated age-standardized excess death rates and ratios from July 2020 to June 2022 for states with weak and strong COVID-19 restrictions, and differences between them. Weak restrictions refer to population-weighted average values of standardized activity limitations scores, standardized mask requirements, mask and vaccination mandates, and state prohibitions on vaccination mandates and mask mandates in schools for the 10 states with the lowest overall restriction scores. Strong restrictions refer to corresponding population-weighted averages for the 10 states with the highest overall restriction scores. These are estimated from population-weighted regressions of the outcomes on these variables and excess death rates or ratios from March 2020 to June 2020 (N = 51). Behavior shows the estimated age-standardized excess death rates and ratios from July 2020 to June 2022 due to behaviors responses associated with weak and strong COVID-19 restrictions. The behaviors include mask use, mobility reductions, and vaccination rates. These are estimated from population-weighted regressions of behaviors on restrictions and of excess death rates and ratios on the corresponding behaviors with additional controls for excess death rates or ratios from March 2020 to June 2020. For calculating marginal effects, estimated values of behaviors from the first set of regressions are used in the second stage. Error bars show 95% CIs based on standard errors.

Of primary interest were predicted effects of packages of strong vs weak restrictions, with the difference between them denoted as the “restriction premium” where negative numbers indicated lower excess death rates or ratios. Strong restrictions were associated with more favorable outcomes, including an excess death rate of 282 per 100 000 over the 2-year period that was 135 per 100 000 (32%) less than the 417 per 100 000 estimated for weak restrictions; similarly, the restriction premium was −0.048 for excess death ratios (0.188 vs 0.236), a 20% difference (Table and Figure 4). The restriction premium was larger, as a percentage of national baseline mortality rates, for excess death rates than ratios (−0.079 vs −0.048) consistent with these 2 estimates bounding the actual effects.

**Table.** Estimated Excess COVID-19 Death Rates, Ratios, and Number of Deaths for States With Weak vs Strong Restrictions, and Percentage of Difference Associated With Behavioral Changes^a^

Outcome	Excess death rate (SE)			Excess death ratio (SE)		
	Weak^b^	Strong^c^	Difference, %	Weak^b^	Strong^c^	Difference, %
Death rate	417.0 (28.1)^d^	282.2 (15.8)^d^	−134.8 (32.7)^d^	NA	NA	NA
Death ratio (SE)	0.244 (0.016)^d^	0.165 (0.009)^a^	−0.079 (0.019)^d^	0.236 (0.012)^d^	0.188 (0.009)^d^	−0.048 (0.016)^d^
Excess deaths, No.	1 384 138 (93 140)	936 746 (52 500)	−447 392 (108 408)	1 337 223 (65 439)	1 066 349 (52 833)	−270 874 (90 308)
Associated with behaviors, %	NA	NA	78.5 (13.1)	NA	NA	49.1 (24.3)

Abbreviations: NA, not applicable; SE, standard error.

^a^ Estimated age-standardized excess death rates (per 100 000) and ratios for July 2020 to June 2022 for states with weak vs strong COVID-19 restrictions. These were estimated from population-weighted regressions of the outcomes on these variables plus age-specific excess death rates or ratios from March 2020 to June 2020 (N = 51). When the death ratio is based on excess death rates, it is calculated as the estimated excess death rate divided by the national prepandemic death rate. The number of excess deaths is for July 2020 to June 2022 and is calculated by multiplying the excess death rate by the national 2021 population divided by 100 000, or the excess death ratio by the annualized baseline death rate and the 2021 population divided by 100 000. Difference indicates estimated difference between strong vs weak restrictions. The percentage related to behaviors was estimated from population-weighted regressions of behaviors on restrictions, and then, of excess death rates and ratios on behaviors as well as excess death rates or ratios from March 2020 to June 2020. Behaviors refer to mask use, mobility reductions, and full vaccination rates. Robust standard errors are shown in parentheses.

^b^ Weak restrictions refer to population-weighted average values of standardized activity limitations scores, standardized mask requirements, mask and vaccination mandates, and state prohibitions on vaccination mandates and mask mandates in schools for the 10 states with the lowest overall restriction scores.

^c^ Strong restrictions refer to corresponding population-weighted averages for the 10 states with the highest overall restriction scores.

^d^ *P* < .01.

These results were robust to sensitivity checks including separately controlling for the 7 specific activity limitations rather than using the composite score; incorporating the overall restriction score as the single policy covariate; excluding the control for the initial pandemic period (March 2020 to June 2020) excess death rates or ratios; estimating the model over the full pandemic period (March 2020 to June 2022) with Connecticut, Massachusetts, New Jersey, and New York excluded; and using COVID-19 mortality, rather than all excess deaths, as the dependent variable (eTable 8 in Supplement 1). Larger restriction effects were also observed in the second analysis year (July 2021- June 2022) than in the first (July 2020 to June 2021).

Using the primary specifications, the estimates suggested that if all states had weak restrictions, there would have been 1.3 million to 1.4 million excess deaths from July 2020 to June 2022, that is, 271 000 to 447 000 more than estimated with universal strong restrictions, a 25% to 48% difference (Table). Compared to the 1.18 million actual excess deaths over the study period, weak restrictions in all states would have resulted in an estimated 13% to 17% increase, and strong restrictions in a 10% to 21% decrease. Behavioral changes were associated with 49% to 79% of the overall restriction premia (Table and Figure 4; eTable 9 in Supplement 1).

## Discussion

COVID-19 restrictions were associated with substantial reductions in excess pandemic deaths in the US. If all states had weak restrictions, as defined in the Methods section, estimated excess deaths from July 2020 to June 2022 would have been 25% to 48% higher than if all had imposed strong restrictions. Behavioral responses provided a potentially important mechanism for this, being associated with 49% to 78% of the overall difference.

This study’s methods departed from prior related research^20,21,40,41^ in 2 particularly important ways. First, the analysis started in July 2020, 4 months after the first substantial COVID-19 fatalities in the US. There was little policy variation during these months, and deaths during this period were probably largely idiosyncratic—reflecting factors such as initial infections in areas that were travel destinations—and so were not strongly reflective of these policies, which also likely impacted mortality with a delay. This is important given that states with high initial mortality rates adopted or retained relatively restrictive policies and had low subsequent excess death rates. Second, while earlier studies used various methods of controlling for potential confounding factors, these efforts were almost certainly incomplete, leaving open the possibility of considerable omitted variables bias. This was partially addressed by examining excess death rates and ratios, which provided likely upper and lower bounds on the restriction effects.

These study findings do not support the views of those opposing COVID-19 restrictions who erroneously believe the restrictions did not work. To the contrary, the package of policies implemented by some states probably saved many lives. However, all restrictions were not equally successful. Evidence of protective effects was weakest for activity limitations. This may have partially concealed benefits at the start of the pandemic, before the analysis period, or reflected reverse causation given that most activity limitations were implemented before political considerations dominated, when states were likely to be especially responsive to local variations in infection rates.

Mortality reductions were not necessarily sufficient to justify imposing restrictions because they also imposed a variety of costs. Some of these, such as the “loss of liberty,” reflect normative beliefs that are difficult to precisely value. However, using value of statistical life estimates ranging from $4.7 million to $11.6 million,^42,43^ the estimated lives saved from strong (vs weak) restrictions over the 2-year period were worth $1.3 trillion to $5.2 trillion—6% to 22% of 2021 gross domestic product—providing a possible benchmark against which to evaluate this loss. Supplement 1 details these calculations.

Some harms were specific and potentially more measurable. Prior research indicates that school closings hurt educational outcomes.^44,45,46^ The absence of evidence that these also reduced pandemic deaths suggests that they may have been too aggressively pursued in some states. On the other hand, school mask mandates were probably more effective and imposed lower costs. Similarly, the social isolation experienced by nursing home residents was harmful,^47^ raising questions about activity limitations that restricted personal interactions for this group.

### Limitations

This study had limitations. First, even with its methodological innovations, the cross-sectional design makes it difficult to identify causation. Second, with only 51 observations (1 for each state plus the District of Columbia), the data were not robust enough to include controls for other state demographic or policy characteristics, although these effects were partially accounted for, to the extent that they influenced baseline mortality. Third, although the restrictions examined were more comprehensive than in prior analyses, they were incomplete in a variety of ways. For example, they did not account for restrictions imposed at the substate level (eg, by cities and school districts). The resulting measurement error may have biased the policy effects toward zero. Conversely, they may have captured the effects of related policies that were not controlled for (eg, state or school vaccine requirements may have been correlated with other types of vaccine mandates in the state). Similarly, data were not available on the exact timing of some policies, requiring the use of dummy variables rather than more precise measures. This also precluded examination of the importance of the specific timing of policy implementation. Fourth, a limited set of behaviors was analyzed. Some of these were self-reported, potentially with error, and they may also have been correlated with omitted factors affecting policy responses in the state. Fifth, the calculation of excess deaths was inexact, although qualitatively similar results were obtained when focusing on fatalities with COVID-19 as the underlying cause. Lastly, state-level policies that were not directly related to the pandemic were not considered, except to the extent these affected baseline death rates.

## Conclusions

 This cross-sectional analysis suggests that strong COVID-19 restrictions saved lives and that the death toll was probably considerably higher than it would otherwise have been in states that resisted imposing these restrictions, banned their use, or implemented them for only relatively short periods of time. These findings may be relevant for public health approaches addressing future pandemics and provide methodological approaches that may be useful for calculating excess deaths in a variety of situations.

## Notes

Supplement 1. eAppendix 1. Additional Data and Methods eAppendix 2. Additional Results eFigure 1. Monthly National Excess Death Rates eFigure 2. Monthly Excess Death Rates and Ratios for States with Below and Above Median Restriction Scores, With and Without CT, MA, NJ, and NY eFigure 3. Comparison of Activity Limitation Score with MultiState and WalletHub Mandate Scores eTable 1. Sample Means for States with Strong and Weak Restrictions eTable 2. COVID-19 Deaths and Death Ratios: March 2020 - May 2020, by State eTable 3. COVID-19 Restrictions and Behaviors by State eTable 4. Age-standardized Excess and COVID-19 Death Rates and Ratios eTable 5. Estimated Effect of Single COVID-19 Restrictions on Age-Standardized Excess Death Rates, Ratios, and Behaviors eTable 6. Estimated Effect of Specific Activity Limitations on Age-Standardized Excess Death Rates and Ratios eTable 7. Estimated Effect of Single Behaviors on Age-Standardized Excess Death Rates or Ratios eTable 8. Robustness Checks for Strong vs. Weak Restriction State Differences in Age-Standardized Excess Death Rates and Ratios eTable 9. Estimated Age-Standardized Excess Death Rates and Ratios for States with Weak and Strong COVID-19 Restrictions eTable 10. Estimated Behaviors in States with Weak and Strong COVID-19 Restrictions eTable 11. State to US Mortality Rate Ratios and Selected State Characteristics eTable 12. Components of Alternative Activity Limitations Measures

Supplement 2. Data Sharing Statement

## References

[1] Wallace-Wells D. The Myth of Early Pandemic Polarization. New York Times. Published June 28, 2023. Accessed September 3, 2023. https://www.nytimes.com/2023/06/28/opinion/covid-pandemic-2020-or-covid-pandemic-politics.html

[2] Kulldorrf M, Gupta S, Bhattacharya J. Great Barrington Declaration and Petition. Published 2020. Accessed September 3, 2023. https://gbdeclaration.org/

[3] The Norfolk Group. Questions for a COVID-19 Commission. Published 2023. Accessed September 3, 2023. https://www.norfolkgroup.org

[4] Weber L, Barry-Jester AM. Over Half of States Have Rolled Back Public Health Powers in Pandemic. KFF Health News. Published September 15, 2021. Accessed September 3, 2023. https://kffhealthnews.org/news/article/over-half-of-states-have-rolled-back-public-health-powers-in-pandemic/

[5] Woolf SH. The growing influence of state governments on population health in the United States. JAMA. 2022;327(14):1331-1332. doi:10.1001/jama.2022.3785 35275203 PMC9745671

[6] Chu DK, Akl EA, Duda S, Solo K, Yaacoub S, Schünemann HJ; COVID-19 Systematic Urgent Review Group Effort (SURGE) study authors. Physical distancing, face masks, and eye protection to prevent person-to-person transmission of SARS-CoV-2 and COVID-19: a systematic review and meta-analysis. Lancet. 2020;395(10242):1973-1987. doi:10.1016/S0140-6736(20)31142-9 32497510 PMC7263814

[7] Talic S, Shah S, Wild H, . Effectiveness of public health measures in reducing the incidence of covid-19, SARS-CoV-2 transmission, and covid-19 mortality: systematic review and meta-analysis. BMJ. 2021;375:e068302. doi:10.1136/bmj-2021-068302 34789505 PMC9423125

[8] Zheng C, Shao W, Chen X, Zhang B, Wang G, Zhang W. Real-world effectiveness of COVID-19 vaccines: a literature review and meta-analysis. Int J Infect Dis. 2022;114:252-260. doi:10.1016/j.ijid.2021.11.009 34800687 PMC8595975

[9] Courtemanche C, Garuccio J, Le A, Pinkston J, Yelowitz A. Strong social distancing measures in the United States reduced the COVID-19 growth rate. Health Aff (Millwood). 2020;39(7):1237-1246. doi:10.1377/hlthaff.2020.00608 32407171

[10] Dave D, Friedson AI, Matsuzawa K, Sabia JJ. When do shelter-in-place orders fight COVID-19 best? policy heterogeneity across states and adoption time. Econ Inq. 2021;59(1):29-52. doi:10.1111/ecin.12944 32836519 PMC7436765

[11] Atkeson A. The impact of vaccines and behavior on U.S. cumulative deaths from COVID-19. National Bureau of Economic Research. Published online August 2023. https://www.nber.org/system/files/working_papers/w31525/w31525.pdf

[12] Allen DW. COVID-19 lockdown cost/benefits: a critical assessment of the literature. Int J Econ Bus. 2022;29(1):1-32. doi:10.1080/13571516.2021.1976051

[13] Haber NA, Clarke-Deelder E, Feller A, . Problems with evidence assessment in COVID-19 health policy impact evaluation: a systematic review of study design and evidence strength. BMJ Open. 2022;12(1):e053820. doi:10.1136/bmjopen-2021-053820 35017250 PMC8753111

[14] Chetty R, Stepner M, Abraham S, . The association between income and life expectancy in the United States, 2001-2014. JAMA. 2016;315(16):1750-1766. doi:10.1001/jama.2016.4226 27063997 PMC4866586

[15] Montez JK, Beckfield J, Cooney JK, . US state policies, politics, and life expectancy. Milbank Q. 2020;98(3):668-699. doi:10.1111/1468-0009.12469 32748998 PMC7482386

[16] Harris JE. Critical role of the subways in the initial spread of SARS-CoV-2 in New York City. Front Public Health. 2021;9:754767. doi:10.3389/fpubh.2021.754767 35004575 PMC8733200

[17] Desmet K, Wacziarg R. JUE insight: understanding spatial variation in COVID-19 across the United States. J Urban Econ. 2022;127:103332. doi:10.1016/j.jue.2021.103332 33723466 PMC7948676

[18] Prince JT, Simon DH. The effect of international travel on the spread of COVID-19 in the United States. South Econ J. 2023;90(2):224-241. doi:10.1002/soej.12661

[19] Horita N, Fukumoto T. Global case fatality rate from COVID-19 has decreased by 96.8% during 2.5 years of the pandemic. J Med Virol. 2023;95(1):e28231. doi:10.1002/jmv.28231 36253938 PMC9874414

[20] Kerpen P, Moore S, Mulligan CB. A final report card on the states’ response to COVID-19. Int J Econ Bus. 2023;30(2):139-158. doi:10.1080/13571516.2023.2176636

[21] Bollyky TJ, Castro E, Aravkin AY, . Assessing COVID-19 pandemic policies and behaviours and their economic and educational trade-offs across US states from Jan 1, 2020, to July 31, 2022: an observational analysis. Lancet. 2023;401(10385):1341-1360. doi:10.1016/S0140-6736(23)00461-0 36966780 PMC10036128

[22] Saletta M, Saletta M, Ho V. State Restrictions and the COVID-19 Death Rate. Rice University, Baker Institute for Public Policy; 2020. Accessed March 10, 2023. https://www.bakerinstitute.org/research/state-restrictions-and-covid-19-death-rate

[23] von Elm E, Altman DG, Egger M, Pocock SJ, Gøtzsche PC, Vandenbroucke JP; STROBE Initiative. The Strengthening the Reporting of Observational Studies in Epidemiology (STROBE) statement: guidelines for reporting observational studies. Lancet. 2007;370(9596):1453-1457. doi:10.1016/S0140-6736(07)61602-X 18064739

[24] US Centers for Disease Control and Prevention. Bridged-Race Population Estimates. Published 2023. Accessed March 10, 2023. https://wonder.cdc.gov/bridged-race-population.html

[25] US Centers for Disease Control and Prevention. Multiple Cause of Death Data 1999-2022. Published 2024. https://wonder.cdc.gov/wonder/help/mcd.html

[26] Woolf SH, Chapman DA, Sabo RT, Zimmerman EB. Excess deaths from COVID-19 and other causes in the US, March 1, 2020, to January 2, 2021. JAMA. 2021;325(17):1786-1789. doi:10.1001/jama.2021.5199 33797550 PMC8019132

[27] Rossen LM, Branum AM, Ahmad FB, Sutton PD, Anderson RN. Notes from the field: update on excess deaths associated with the COVID-19 pandemic: United States, January 26, 2020-February 27, 2021. MMWR Morb Mortal Wkly Rep. 2021;70(15):570-571. doi:10.15585/mmwr.mm7015a4 33857065 PMC8344999

[28] Sanmarchi F, Golinelli D, Lenzi J, . Exploring the gap between excess mortality and COVID-19 deaths in 67 countries. JAMA Netw Open. 2021;4(7):e2117359. doi:10.1001/jamanetworkopen.2021.17359 34269809 PMC8285734

[29] Wang H, Paulson KR, Pease SA, ; COVID-19 Excess Mortality Collaborators. Estimating excess mortality due to the COVID-19 pandemic: a systematic analysis of COVID-19-related mortality, 2020-21. Lancet. 2022;399(10334):1513-1536. doi:10.1016/S0140-6736(21)02796-3 35279232 PMC8912932

[30] Ruhm CJ. Excess deaths in the United States during the first year of COVID-19. Prev Med. 2022;162:107174. doi:10.1016/j.ypmed.2022.107174 35878708 PMC9304075

[31] Ruhm CJ. Pandemic and recession effects on mortality in the US during the first year of COVID-19. Health Aff (Millwood). 2022;41(11):1550-1558. doi:10.1377/hlthaff.2022.00364 36343324

[32] Ruhm CJ. The evolution of excess deaths in the United States during the first 2 years of the COVID-19 pandemic. Am J Epidemiol. 2023;192(12):1949-1959. doi:10.1093/aje/kwad127 37222463 PMC10988222

[33] Grabowski DC, Mor V. Nursing home care in crisis in the wake of COVID-19. JAMA. 2020;324(1):23-24. doi:10.1001/jama.2020.8524 32442303

[34] Institute for Health Metrics and Evaluation. COVID-19 Database. Institute for Health Metrics and Evaluation. Published 2023. Accessed April 18, 2023. https://www.healthdata.org/covid

[35] Ballotpedia: The Encyclopedia of American Politics. State-level mask requirements in response to the coronavirus (COVID-19) pandemic, 2020-2022. Ballotpedia. Published 2023. Accessed May 19, 2023. https://ballotpedia.org/State-level_mask_requirements_in_response_to_the_coronavirus_(COVID-19)_pandemic,_2020-2022

[36] Kaiser Family Foundation. State COVID-19 Data and Policy Actions. Published February 10, 2022. Accessed May 19, 2023. https://www.kff.org/report-section/state-covid-19-data-and-policy-actions-policy-actions/

[37] US Centers for Disease Control and Prevention. COVID-19 Vaccination Trends in the United States, National and Jurisdictional. Published 2023. Accessed March 10, 2023. https://data.cdc.gov/Vaccinations/COVID-19-Vaccination-Trends-in-the-United-States-N/rh2h-3yt2

[38] Opportunity Insights. The Economic Tracker. Published 2023. Accessed April 18, 2023. https://tracktherecovery.org/

[39] Raifman J, Nocka K, Jones D, . COVID-19 US state policy database (CUSP). Published 2023. Accessed September 21, 2023. http://www.tinyurl.com/statepolicies

[40] Feldman JM, Bassett MT. Variation in COVID-19 mortality in the US by race and ethnicity and educational attainment. JAMA Netw Open. 2021;4(11):e2135967. doi:10.1001/jamanetworkopen.2021.35967 34812846 PMC8611482

[41] Gearhart R, Michieka N, Anders A. The effectiveness of COVID deaths to COVID policies: a robust conditional approach. Econ Anal Policy. 2023;79:376-394. doi:10.1016/j.eap.2023.06.026 37363405 PMC10276656

[42] Viscusi WK. Best estimate selection bias in the value of a statistical life. J Benefit Cost Anal. 2018;9(2):205-246. doi:10.1017/bca.2017.21

[43] Robinson LA, Sullivan R, Shogren JF. Do the benefits of COVID-19 policies exceed the costs? exploring uncertainties in the age-VSL relationship. Risk Anal. 2021;41(5):761-770. doi:10.1111/risa.13561 32677076 PMC7405126

[44] Goldhaber D, Kane T, McEachin A, Morton E, Patterson T, Staiger D. The Consequences of Remote and Hybrid Instruction During the Pandemic. National Bureau of Economic Research; 2022. doi:10.3386/w30010

[45] Jack R, Halloran C, Okun J, Oster E. Pandemic schooling mode and student test scores: evidence from US school districts. Am Econ Rev Insights. 2023;5(2):173-190. doi:10.1257/aeri.20210748

[46] Jack R, Oster E. COVID-19, school closures, and outcomes. J Econ Perspect. 2023;37(4):51-70. doi:10.1257/jep.37.4.51

[47] Cronin CJ, Evans WN. Nursing home quality, COVID-19 deaths, and excess mortality. J Health Econ. 2022;82:102592. doi:10.1016/j.jhealeco.2022.102592 35104669 PMC8776351

