# Supplementary material for: US State Restrictions and Excess COVID-19 Pandemic Deaths
Source: JAMA Health Forum. 2024 Jul 26;5(7):e242006. doi: 10.1001/jamahealthforum.2024.2006 (PMC11282449; doi:10.1001/jamahealthforum.2024.2006)
Supplement: Supplement 1. — eAppendix 1. Additional Data and Methods eAppendix 2. Additional Results eFigure 1. Monthly National Excess Death Rates eFigure 2. Monthly Excess Death Rates and Ratios for States with Below and Above Median Restriction Scores, With and Without CT, MA, NJ, and NY eFigure 3. Comparison of Activity Limitation Score with MultiState and WalletHub Mandate Scores eTable 1. Sample Means for States with Strong and Weak Restrictions eTable 2. COVID-19 Deaths and Death Ratios: March 2020 - May 2020, by State eTable 3. COVID-19 Restrictions and Behaviors by State eTable 4. Age-standardized Excess and COVID-19 Death Rates and Ratios eTable 5. Estimated Effect of Single COVID-19 Restrictions on Age-Standardized Excess Death Rates, Ratios, and Behaviors eTable 6. Estimated Effect of Specific Activity Limitations on Age-Standardized Excess Death Rates and Ratios eTable 7. Estimated Effect of Single Behaviors on Age-Standardized Excess Death Rates or Ratios eTable 8. Robustness Checks for Strong vs. Weak Restriction State Differences in Age-Standardized Excess Death Rates and Ratios eTable 9. Estimated Age-Standardized Excess Death Rates and Ratios for States with Weak and Strong COVID-19 Restrictions eTable 10. Estimated Behaviors in States with Weak and Strong COVID-19 Restrictions eTable 11. State to US Mortality Rate Ratios and Selected State Characteristics eTable 12. Components of Alternative Activity Limitations Measures [file jamahealthforum-e242006-s001.pdf]

Supplemental Online Content

Ruhm CJ. US State Restrictions and Excess COVID-19 Pandemic Deaths. *JAMA Health Forum.* Published online July 26, 2024. doi:10.1001/jamahealthforum.2024.2006

eAppendix 1. Additional Data and Methods

eAppendix 2. Additional Results

eFigure 1. Monthly National Excess Death Rates

eFigure 2. Monthly Excess Death Rates and Ratios for States with Below and Above Median Restriction Scores, With and Without CT, MA, NJ, and NY

eFigure 3. Comparison of Activity Limitation Score with MultiState and WalletHub Mandate Scores

eTable 1. Sample Means for States with Strong and Weak Restrictions

eTable 2. COVID-19 Deaths and Death Ratios: March 2020 - May 2020, by State

eTable 3. COVID-19 Restrictions and Behaviors by State

eTable 4. Age-standardized Excess and COVID-19 Death Rates and Ratios

eTable 5. Estimated Effect of Single COVID-19 Restrictions on Age-Standardized Excess Death Rates, Ratios, and Behaviors

eTable 6. Estimated Effect of Specific Activity Limitations on Age-Standardized Excess Death Rates and Ratios

eTable 7. Estimated Effect of Single Behaviors on Age-Standardized Excess Death Rates or Ratios

eTable 8. Robustness Checks for Strong vs. Weak Restriction State Differences in Age-Standardized Excess Death Rates and Ratios

eTable 9. Estimated Age-Standardized Excess Death Rates and Ratios for States with Weak and Strong COVID-19 Restrictions

eTable 10. Estimated Behaviors in States with Weak and Strong COVID-19 Restrictions

eTable 11. State to US Mortality Rate Ratios and Selected State Characteristics

eTable 12. Components of Alternative Activity Limitations Measures

This supplemental material has been provided by the authors to give readers additional information about their work.

eAppendix 1. Additional Data and Methods

<u>*Data*</u>

Population

Most estimates were weighted by July 1st 2020 resident state populations, obtained from bridged-race population estimates available on *CDC Wonder*.[1] The denominators for death rates during the pandemic were corresponding 2021 populations, using data from the same source.

Deaths

Mortality data came from the CDC *Multiple Cause of Death* data available on CDC Wonder.[2] Deaths from COVID-19 were those with underlying cause of death code U07.1. Deaths counts were converted to rates per 100,000 using 2021 state populations. Excess death rates were calculated using procedures described below, with baseline mortality data in the primary specifications from 7/17-6/19, which corresponds to the same period as in the main analysis but three years before it. COVID-19 and excess death rates were collected separately for persons aged <45, 45-64, 65-84, and ≥85, and these were used to compute age-standardized rates for the full population and those <65 and ≥65, as detailed in the Methods section below.

COVID-19 Restrictions

Primary information on state activity limitations came from the Institute for Health Metrics and Evaluation (IHME). These data were used in a *Lancet* article[3] and were graciously provided to me by Emma Castro & Joseph Dieleman; they are also available through IHME's COVID-19 database.[4] Activity limitations included: stay-at-home orders, restaurant closures, bar closures, gym/pool/leisure activity closures, public gatherings (≥50 people indoors or ≥100 people outdoors), and mandated closures for in-person learning in primary schools and institutions of higher education. In each case, the percentage of days the mandate was in effect was calculated from 7/20-6/22. The population-weighted average of these seven measures was also computed as an overall activity limitations measure. Alternative sources of activity limitations were also examined and are shown below to have similar patterns to those used here. These are discussed below.

Most states required individuals to wear masks when in public for portions of the pandemic. The percentage of the analysis period these were in effect was calculated using data from Ballotpedia,[5] except for Washington DC, where the mask mandate data were from IMHE.[4]

Additional data on COVID-19 related mask and vaccine mandates were incorporated including whether state government or school employees were required to or prohibited from being required to be vaccinated as of February 2022, and whether there were mask requirements in schools. The details of state vaccination mandates varied. Typically, the requirement was that state and local government employees be vaccinated or subjected to regular testing. Sometimes the vaccination mandates covered other groups, such as healthcare workers or staff in some health care facilities. School vaccination mandates usually referred to teachers and staff in primary and secondary schools during the 2021-2022 academic year, while mask mandates referred to students, staff, teachers, and visitors. Vaccination prohibitions generally outlawed proof of vaccination as a condition of employment but were also sometimes broader, including banning requiring vaccinations as a condition for entering a business. School mask mandate prohibitions allowed parents to opt out of mask mandates for their children in schools or sometimes completely disallowed school-related mask mandates. Data on these policies came from the Kaiser Family Foundation.[6]

Vaccinations

State and national vaccination data were obtained from the Centers for Disease Control and Prevention,[7] which provided daily information on numbers and rates of vaccinations by state and for the entire country. Data from administrative records were retained for Sunday of each week beginning December 13, 2020, the week of the first vaccinations. The variable focused upon here was the percentage of the population that had completed a primary COVID-19 vaccination series, defined as having received one dose of the Johnson & Johnson vaccine or two doses of the Pfizer-BioNTech or Moderna vaccines.

Other Behaviors

In addition to vaccination rates, two other behaviors were analyzed. The first was the percentage of days, from 7/20-6/22 that individuals stated that they always wore a mask when

outside their home, referred to as “mask use”. The second was the reduction in time spent outside the home, compared to the pre-pandemic period, referred to as “mobility reductions”. The mask-use data came from the IHME COVID-19 database.[4] Time outside of home data was from Google *COVID-19 Community Mobility Reports*,[8] as compiled by Chetty et al.[9] and obtained from the Opportunity Insights *Economic Tracker*.[10]

Methods

Since the risk of dying from COVID-19 and related causes increased dramatically with age, age-standardized mortality rates were calculated by first computing national 2020-year population shares for four age groups (<45, 45-64, 65-84, ≥85) and then calculating age-standardized the mortality rate, M_i^{age}, as:

$$M_i^{age} = \sum_j S_j M_{ij} \quad (1)$$

where M_{ij} was the mortality rate in state i for age group j ($j \epsilon \{< 45,\ 45-64, 65-84, \geq 85\}$) and S_j was the 2020 share of the national population in that age group. Age-specific mortality rates were calculated as the death count in the state and age-group divided by the state’s 2021 age-specific population. Age-standardization reduced the death rates of states with relatively old populations and raised those with young ones.

Pandemic deaths due to COVID-19 or other causes could have been influenced by a variety of factors, such as differences in educational attainment, the industrial composition of employment, and the policy environment. Although some of these could potentially have been directly controlled for, others would likely not have been, resulting in omitted variables biases. To address this, and to account for increases or decreases in deaths from other causes during the pandemic, excess death rates and ratios were calculated. Excess deaths account for mortality directly due to COVID-19 as well as other deaths related to the pandemic but without COVID-19 as the underlying cause, and have been widely used in the literature for this purpose.[11–17]

To compute excess deaths, baseline death rates in state i for age group j, B_{ij}, were first calculated as the state age-specific all-cause death rate over the 7/17–6/19 period. A two-year average was used to conform to the analysis period and to reduce idiosyncratic differences in a

© 2024 Ruhm CJ. *JAMA Health Forum*.

single year. Age-specific excess death rates, E_{ij}, were then computed as the difference between actual death rates over the two-year analysis period (7/20-6/22), M_{ij} and baseline annual death rates from 7/17–6/19,

$$E_{ij} = M_{ij} - B_{ij}. \tag{2}$$

This was appropriate because there was little evidence of consistent trend changes in death rate rates over this period. These were transformed into the age-standardized excess death rates described above as:

$$E_i^{age} = \sum_j S_j \, E_{ij}. \tag{3}$$

Excess death rates accounted for disparities in *levels* of baseline mortality rates across states, to the extent these influences persisted in the same way during the pandemic as before it. This included the potential confounding factors mentioned above as well as many others. [a]

However, because death rates rose during the pandemic, such adjustments in levels may have been insufficient, since equal percentage changes would have raised death rates by larger absolute amounts in states with high than low pre-pandemic mortality rates. If so, and states with lower pre-pandemic death rates were more likely to adopt restrictive policies during the pandemic, using excess death rates as the dependent variable would be likely to have *overstated* the benefits of these restrictions.

An alternative was to focus on excess death ratios computed as:

$$ER_{ij} = (M_{ij}/B_{ij}) - 1 = E_{ij}/B_{ij}, \tag{4}$$

and age-standardized death ratios:

---

[a] Differences in baseline mortality rates partially reflected policy decisions made by states, such as investments in the health infrastructure or in other types of support policies. eTable 11 provides evidence of these differences. For instance, 10 of the 26 states with the highest baseline mortality rates had not expanded Medicaid under the Affordable Care Act by the start of 2020, compared to just 3 of the 25 with the lowest death rates. Monthly TANF benefits for a family of three in 2019, averaged just $337 for the 26 worst performers versus $612 for the 25 best. Other differences could reflect a mix of policies and more exogenous factors. For example, 31% of persons 25 and older had a bachelor's degree in 2020 in the highest 26 mortality rate states versus 37% in the other 25.

$$ER_i^{age} = \sum_j S_j \, ER_{ij}.^{b} \quad (5)$$

These allowed for proportional adjustments in pandemic effects but may have *underestimated* the effectiveness of restrictions if the factors affecting baseline death rates less strongly influenced pandemic deaths, or vice versa. For example, persons living in nursing homes or areas with high population density, or making extensive use of public transportation, may have been particularly vulnerable to COVID-19,[18–20] while these factors were probably closely related to deaths prior to the pandemic. In combination, the analysis of excess death rates and ratios likely bounds the restriction effects. [c]

It is difficult to directly compare the results obtained using excess death rates and ratios, since the units of measurement are different. To facilitate such comparisons, excess death rates were sometimes converted into ratios by dividing them by the pre-pandemic (7/17-6/19) national mortality rates. [d]

Primary regression specifications took the form:

$$D_i^{age} = \alpha D_{i0} + \ Restrict_i \beta + \mu_i, \quad (6)$$

where D_i^{age} was the age-standardized mortality outcome – excess death rates or ratios – measured from 7/20-6/22, D_{i0} was the corresponding excess death rate or ratio from 3/20-6/20, $Restrict$ was a single COVID-19 restriction or a vector of restriction variables, μ was the regression error term, observations were weighted by 2020 year state populations, and confidence intervals were based upon robust standard errors. The sample analyzed included one observation for each of the 50 states plus the District of Columbia (n=51). $\hat{\beta}$ showed the

---

[b] COVID-19 death ratios were calculated in the same way, except that the numerator in the right-hand-side of (4) refers to deaths with COVID-19 as the underlying cause and one is not subtracted from the fraction.

[c] Consider the example of two states with pre-pandemic mortality rates of 400 and 200 per 100,000 respectively. If the pandemic resulted in a 20% increase in mortality in both states, excess death rates in the two states would have been 80 and 40 per 100,000, even though excess ratios in both would have been correctly measured at 0.20. Since states with relatively low baseline mortality rates tended to have strong restrictions, this induced the erroneous evidence of protective effects when using excess mortality rates. At the other extreme, if pandemic mortality were determined by factors unrelated to pre-pandemic mortality rates, the excess death rates would indicate the true effect and the excess death ratios would have understated the protective effects.

[d] Note that these were computed $ER_i = E_i/B_{us}$, where B_{us} was the national baseline mortality rate, which differed from the excess death ratios above, computed as, $ER_i = E_i/B_i$, where B_i was the baseline mortality rate in the state (rather than the entire US).

estimated effect of the specified restriction on the mortality outcome. The continuous restriction variables (activity limitations and general mask requirements) were transformed by subtracting actual values from the national mean and dividing by its standard deviation, so that coefficients on these indicated estimated effects of a one standard deviation change in the variable, often referred to as "effect sizes". Mortality outcomes at the beginning of the pandemic but prior to the analysis period (from 3/20-6/20) were controlled for in case these influenced subsequent excess death rates or ratios as might occur, for example, if early exposure to COVID-19 subsequently provided partial or full immunity.

Coefficients on the individual policy variables were not the major focus (although they were reported in places) due to high multi-collinearity between them. Instead, most emphasis was on estimated values calculated for states with "weak" and "strong" restrictions, and the differences between them. Weak restrictions referred to the population-weighted averages observed for the 10 least restrictive states and strong restrictions to those for the 10 most restrictive states based on an overall state restriction score calculated as:

$$RS_i = AL_i^{std} + MSK_i^{std} + VCST_i + VCSC_i + MSKSC_i - VCPRO_i - MSKPRO_i, \quad (7)$$

where RS was the restriction score, AL^{std} and MSK^{std} referred to the activity limitations score and percent of time that a mask was required outside the home, both standardized to have a population-weighted mean of zero and standard deviation of one, and with $VCST, VCSC, MSKSC, VCPRO$, and $MSKPRO$ indicating dummy variables respectively set to one for vaccination requirements for state employees, school vaccination requirements, school mask requirements, and prohibitions on state vaccination and school mask requirements. This overall restriction score was used to facilitate comparisons, but was not be relied upon in the econometric analysis that followed. However, the state rankings of restriction scores were not sensitive to this particular formulation. For example, the results were similar when giving double weight to the activity limitation score or excluding the two prohibition variables.

The extent to which restriction effects on excess death rates or ratios were associated with behavioral changes was also examined. For this exercise, the total effect of the restrictions was first estimated from equation (6). Next, the corresponding relationships between the restrictions

and the three types of behaviors – mask use, mobility reductions, and vaccination rates – were obtained from estimates of:

$$Behavior_{ik} = Restrict_i \gamma_k + \epsilon_{ik}, \quad (8)$$

where $Behavior_{ik}$ referred to the kth behavior in state i and $\hat{\gamma}_k$ indicated the estimated correlations with restrictions. Once again, estimated differences between states with strong and weak restrictions were calculated. Referring to these as $\gamma_k^{'}$, the overall difference in death outcomes associated with behavioral changes was obtained by regressing excess death rates or ratios on the behaviors:

$$D_i^{age} = Behavior_{ik}\delta_k + \pi_i, \quad (9)$$

and calculating the explained change as:

$$\sum_{k=1}^{3}(\hat{\gamma}_k \times \hat{\delta}_k), \quad (10)$$

where $\hat{\delta}_k$ was the estimated relationship of the behavior to the mortality outcome. This association was then compared to the total effect obtained from equation (6). Note that the estimates are likely to indicate an upper-bound on the actual effects of behavior changes since states imposing strong restrictions may have experienced stronger than average behavioral changes even in their absence.

Value of Lives Saved From Strong Restrictions

The point estimates suggest that strong restrictions in all states would have saved 270,874 (447,392) lives based on excess death ratios (rates), over the two-year analysis period, compared to if all states had weak restrictions. These were valued at between $4.74 and $11.55 million. The $11.55 million was based on Viscusi's[21] median estimate of the values of a statistical life (VSL) of $10.1 million in 2015, adjusted for inflation to 2021 year dollars. [e] Robinson et al.[22] provided three estimates ranging from $4.47 to $10.63 million in 2019 dollars, depending on how VSL was assumed to vary with age of death. Converting these to 2021 dollars, they were equal to $4.74 to $11.27 million. The lowest of these was used here.

[e] Viscusi's average estimate was $2 million higher than the median (used here); using this would provide a larger dollar loss.

Multiplying these values by the estimated reduction in excess deaths lives provided a total estimated value of $1.28 to $5.17 trillion, which was equal to 5.5% to 22.2% of the 2021 GDP of $23.32 trillion. These estimates did not value reductions in morbidity associated with strong restrictions, which are likely to have been substantial.[23]

eAppendix 2. Additional Results

This appendix contains a number of figures and tables, providing supplementary and supporting results to those contained in the table and four figures provided in the text of the main article. These are described here, sometimes with additional discussion.

eFigure 1. Displays monthly excess death rates during 2020-2022 and shows that the time profile is virtually identical to that for counts of deaths with COVID-19 as an underlying cause, presented in Figure 1A.

eFigure 2. Shows monthly excess death rates for subsamples of states with above and below median overall restrictions scores, corresponding to Figures 2E and 2F, as well those with above median scores but excluding the four states (CT, MA, NJ, and NY) that were the most impacted during the pre-analysis period (3/20-6/20). The small difference observed during the 7/20-6/22 period, when these four states are eliminated, indicates that early COVID-19 exposure provided only a minimal subsequent protective effect. This small remaining impact was addressed in the regression analysis by including an early exposure control in the models.

eFigure 3. Shows relationship between the activity limitation scores used in this analysis and alternative measures that are discussed below.

eTable 1. Displays population-weighted mean values of dependent variables, restrictions, and behaviors for all states and the 10 states with the weakest and strongest restrictions, as measured by the overall restriction score. The subsample means for the standardized activity limitations score and mask requirements, as well as for the mandate and prohibition dummy variables, were used to defined “weak” and “strong” restrictions (and the difference between them) utilized in the subsequent analysis.

eTable 2. Shows state number of deaths with COVID-19 as an underlying cause from 3/20-5/20, and corresponding state populations and death-to-population ratios. The last of these provided the inputs for Figure 1B.

eTable 3. Indicates average values of the state restrictions and behaviors for the two-year (7/20-6/22) analysis period.

eTable 4. Shows state excess death rates and ratios over the two-year analysis period, which provided the inputs for Figures 2C and 2D. It also shows the corresponding rate of deaths with COVID-19 as the underlying cause, and the excess death ratio from this cause.

eTable 5. Shows coefficient estimates for the restrictions on excess death rates and ratios, and behaviors, which provided the inputs for Figures 3A-3D. These coefficients were obtained from regressions that included only the single specified restriction, and the pre-analysis value of the dependent variable. The effects of restrictions on behaviors was calculated using the raw values of the dependent variables (magnitudes) and with the outcomes normalized to have a mean of zero and a standard deviation of one.

eTable 6. Shows coefficient estimates for specific activity limitations, rather than for the composite activity limitations score that was used elsewhere.

eTable 7. Displays the coefficient estimates obtained from regressions of excess death rates or ratios on single behaviors. These provided the inputs for Figures 3E and 3F.

eTable 8. Provides robustness checks. The "Main" specification in column (1) was the same as the model detailed in Table 1. Other estimates were largely consistent with those from this specification. The estimated restriction premia were the same or marginally higher when separately including the seven specific activity limitations and somewhat lower when controlling for only the overall restriction score (models 2 and 3). Excluding the control for the initial pandemic surge in deaths modestly increased the restriction premia, consistent with a slight protective effect of early pandemic exposure on later mortality (column 4). The premia were also somewhat lower when including the full pandemic period, 3/20-6/22, but without the four states (CT, MA, NJ, and NY) that were particularly hard-hit for idiosyncratic reasons at

its start (model 5).[f] The restriction premia fell when using COVID-19 deaths as the outcomes, as anticipated since the number of COVID-19 deaths was only around two-thirds of excess deaths (column 6). The premium was larger for Year 2 (7/21-6/22) than Year 1 (7/20-6/21), which makes sense since activity limitations, which were concentrated in Year 1, were largely uncorrelated with deaths, while most of the remaining restrictions were primarily in place in Year 2 (models 7 and 8).

eTable 9. Shows the overall estimated effects of packages of strong versus weak restrictions on excess death rates and ratios, as well as the difference between them. It also shows how much of the overall estimated effect was related to behavioral changes. These provide the inputs to Table 1 and Figure 4.

eTable 10. Indicates relationship of weak versus strong restrictions to behaviors. This was an input used when calculating the restriction effects on excess death rates and ratios that operated through the changes in behaviors described in eTable 9.

eTable 11. Provides additional details on state level mortality rates during the analysis period, as a ratio of pre-pandemic mortality rates, and levels of educational attainment, Medicaid expansion status, or TANF benefit levels. These results indicated that states with weak restrictions also tended to have high pre-pandemic mortality rates and low levels of education and social support policies.

eTable 12. Compares features of the IHME activity limitation data, used in this analysis, to those from two alternative data sources, described below.

eTable 13. Compares activity limitation scores from the IHME activity limitation data to those from two alternative sources, as described below.

Alternative Sources of Activity Limitations Data

Information on state activity limitations and mask mandates related to COVID-19 were obtained from the Institute for Health Metrics and Evaluation (IHME) COVID-19 database.[4] This

[f] Early (3/20 – 6/20) excess death rates were not controlled for in this specification, since these deaths were also included in the dependent variable.

included information on restrictions related to: stay-at-home orders, restaurant closures, bar closures, gym/pool/leisure activity closures, public gatherings (≥50 people indoors or ≥100 people outdoors), and mandated closures for in-person learning in primary schools and institutions of higher education. The population-weighted average of the seven restrictions was also computed as an overall restriction intensity measure.

Two alternative measures of activity limitations were used to check consistency with the measure used here. The first came from MultiState, as part of their *COVID-19 Reopening Guide*. Data published on the website were only for a single point-in-time; however, Bill Kramer, Vice President for Policy at MultiState provided me with supplemental data for other dates, beginning on 5/4/2020 and continuing through 7/8/2021. The data were available at various intervals, ranging from daily to approximately bi-monthly. For this project, data were used for periods roughly corresponding to the beginning and middle of each month. Specifically, data were used in 2020 for 5/4, 5/16, 6/1, 6/16, 7/1, 7/16, 7/31, 8/17, 8/31, 9/15, 10/1, 10/16, 11/2, 11/16, 12/1, 12/17, and 12/30 of 2020; and in 2021 for 1/18, 2/3, 2/16, 3/2, 3/15, 4/1, 4/15, 4/30, 5/17, 6/1, 6/15, and 7/1. For each of the 50 states (Washington D.C. was excluded) the data were coded on a scale of 0 to 100, where 0 was most restrictive (least open) and 100 was least restrictive (most open). They were reverse-coded for this project so that 0 is least restrictive and 100 most restrictive.

Components of the score included stay-at-home orders, and restrictions on opening or operating: non-essential (not consumer facing) offices, non-essential retail businesses, personal care services, physical fitness businesses, restaurants (beyond pickup and delivery), bars (beyond pickup and delivery), venues that serviced large crowds (>50 people), and state preemption of more restrictive local measures. In addition, before 2/8/2021, measures of the definition of state essential businesses and whether or not construction sites were allowed to operate were included. On that date, these were replaced by indicators for state mandatory curfews and restrictions on private or public gatherings. This change probably increased the restrictiveness score by around 4 points. The main variable used here was the average score over the 29 time periods. In addition, average scores were separately computed for measured periods during calendar years 2020 and 2021.

Data from WalletHub provided second source of supplemental information on activity restrictions. These data were also scored on a 0 to 100 basis, with 0 (100) originally referring to the most (least) restrictive, and they were again reverse scored here so that higher scores indicated greater restrictions. Data for 51 jurisdictions (the 50 states plus Washington D.C.) were obtained for the following dates in 2000: 5/5, 5/19, 6/9, 6/23, 7/7, 7/21, 8/11, 9/15, and 10/5, with additional 2021 information available for 1/26, 3/2, and 4/5. The 2020 data came from a Rice University research report,[24] the 4/5/21 data from a WalletHub report,[25] and the 1/6/21 and 3/2/21 data were directly provided to me by Diana Polk, Communications Manager at WalletHub.

The WalletHub scores were based on 17 components related to: reopening of bars and restaurants, restrictions on local gatherings, strictness of shelter-in-place orders, business immunity from COVID-19 claims, reopening of non-essential businesses, public mask requirements, state court closures/suspensions, statewide school closures, work-from-home requirements & regulations, mandated contact tracing, multistate reopening agreements, state guidance on consumer health checks at restaurants, travel restrictions, penalties for COVID-19 legislation non-compliance, guidance for elective medical procedures, guidance for assisted living facilities, and workplace temperature screenings. These factors were differentially weighted to vary with the severity of the restrictions. For instance, the first three restrictions listed above accounted for 36% of the total score and the first nine for 79% of it. As with the MultiState data, average scores over all available periods were computed, as were separate averages for 2020 and 2021 calendar year observations.

As shown on eTable 12, the scoring systems differed somewhat, with the MultiState scores emphasizing restrictions on leaving home and business operations, while the WalletHub scores were somewhat broader, including factors such as school closures, mask requirements and other restrictions. The main, IHME measure was a mix of both. The composite scores are nonetheless highly correlated, with R-Squared between the population-weighted IHME and MultiState (WalletHub) scores being 0.803 (0.760). (The correlation between the population-weighted MultiState and WalletHub scores is 0.848.) See eFigure 3 for additional details.

eReferences

1. Centers for Disease Control and Prevention. Bridged-Race Population Estimates [Internet]. 2023 [cited 2023 Mar 10];Available from: https://wonder.cdc.gov/bridged-race-population.html

2. Centers for Disease Control and Prevention. Multiple Cause of Death Data 1999-2022 [Internet]. 2024;Available from: https://wonder.cdc.gov/wonder/help/mcd.html

3. Bollyky TJ, Castro E, Aravkin AY, et al. Assessing COVID-19 pandemic policies and behaviours and their economic and educational trade-offs across US states from Jan 1, 2020, to July 31, 2022: an observational analysis. Lancet 2023;S0140-6736(23)00461-0.

4. Institute for Health Metrics and Evaluation. COVID-19 Database [Internet]. Institute for Health Metrics and Evaluation. 2023 [cited 2023 Apr 18];Available from: https://www.healthdata.org/covid

5. Ballotpedia: The Encyclopedia of American Politics. State-level mask requirements in response to the coronavirus (COVID-19) pandemic, 2020-2022 [Internet]. Ballotpedia. 2023 [cited 2023 May 19];Available from: https://ballotpedia.org/State-level_mask_requirements_in_response_to_the_coronavirus_(COVID-19)_pandemic,_2020-2022

6. Kaiser Family Foundation. State COVID-19 Data and Policy Actions [Internet]. 2022 [cited 2023 May 19];Available from: https://www.kff.org/report-section/state-covid-19-data-and-policy-actions-policy-actions/

7. Centers for Disease Control and Prevention. COVID-19 Vaccination Trends in the United States, National and Jurisdictional [Internet]. 2023 [cited 2023 Mar 10];Available from: https://data.cdc.gov/Vaccinations/COVID-19-Vaccination-Trends-in-the-United-States-N/rh2h-3yt2

8. Google. COVID-19 Community Mobility Reports [Internet]. COVID-19 Community Mobility Report. 2022 [cited 2023 Apr 18];Available from: https://www.google.com/covid19/mobility?hl=en

9. Chetty R, Friedman JN, Stepner M. The economic impacts of COVID-19: Evidence from a new public database built using private sector data. Opportunity Insights; 2023.

10. Opportunity Insights. The Economic Tracker [Internet]. 2023 [cited 2023 Apr 18];Available from: https://tracktherecovery.org/

11. Woolf SH, Chapman DA, Sabo RT, Zimmerman EB. Excess Deaths From COVID-19 and Other Causes in the US, March 1, 2020, to January 2, 2021. JAMA 2021;

12. Rossen LM, Branum AM, Ahmad FB, Sutton PD, Anderson RN. Notes from the Field: Update on Excess Deaths Associated with the COVID-19 Pandemic - United States, January 26, 2020-February 27, 2021. MMWR Morb Mortal Wkly Rep 2021;70(15):570–1.

13. Sanmarchi F, Golinelli D, Lenzi J, et al. Exploring the Gap Between Excess Mortality and COVID-19 Deaths in 67 Countries. JAMA Netw Open 2021;4(7):e2117359.

14. Wang H, Paulson KR, Pease SA, et al. Estimating excess mortality due to the COVID-19 pandemic: a systematic analysis of COVID-19-related mortality, 2020–21. The Lancet 2022;399(10334):1513–36.

15. Ruhm CJ. Excess deaths in the United States during the first year of COVID-19. Prev Med 2022;162:107174.

16. Ruhm CJ. Pandemic And Recession Effects On Mortality In The US During The First Year Of COVID-19. Health Aff (Millwood) 2022;41(11):1550–8.

17. Ruhm CJ. The Evolution of Excess Deaths in the United States During the First Two Years of the COVID-19 Pandemic. American Journal of Epidemiology 2023;kwad127.

18. Grabowski DC, Mor V. Nursing Home Care in Crisis in the Wake of COVID-19. JAMA 2020;324(1):23.

19. Wong DWS, Li Y. Spreading of COVID-19: Density matters. PLoS ONE 2020;15(12):e0242398.

20. Desmet K, Wacziarg R. JUE Insight: Understanding spatial variation in COVID-19 across the United States. Journal of Urban Economics 2022;127:103332.

21. Viscusi WK. Best Estimate Selection Bias in the Value of a Statistical Life. J Benefit Cost Anal 2018;9(2):205–46.

22. Robinson LA, Sullivan R, Shogren JF. Do the Benefits of COVID-19 Policies Exceed the Costs? Exploring Uncertainties in the Age–VSL Relationship. Risk Analysis 2021;41(5):761–70.

23. Cutler DM, Summers LH. The COVID-19 Pandemic and the $16 Trillion Virus. JAMA 2020;324(15):1495.

24. Saletta M, Saletta M, Ho V. State Restrictions and the COVID-19 Death Rate [Internet]. Rice University, Baker Institute for Public Policy; 2020 [cited 2023 Mar 10]. Available from: https://www.bakerinstitute.org/research/state-restrictions-and-covid-19-death-rate

25. McCann A. States with the Fewest Coronavirus Restrictions [Internet]. WalletHub. 2021 [cited 2023 Mar 10];Available from: https://wallethub.com/edu/states-coronavirus-restrictions/73818

© 2024 Ruhm CJ. *JAMA Health Forum*.

eFigure 1. Monthly National Excess Death Rates

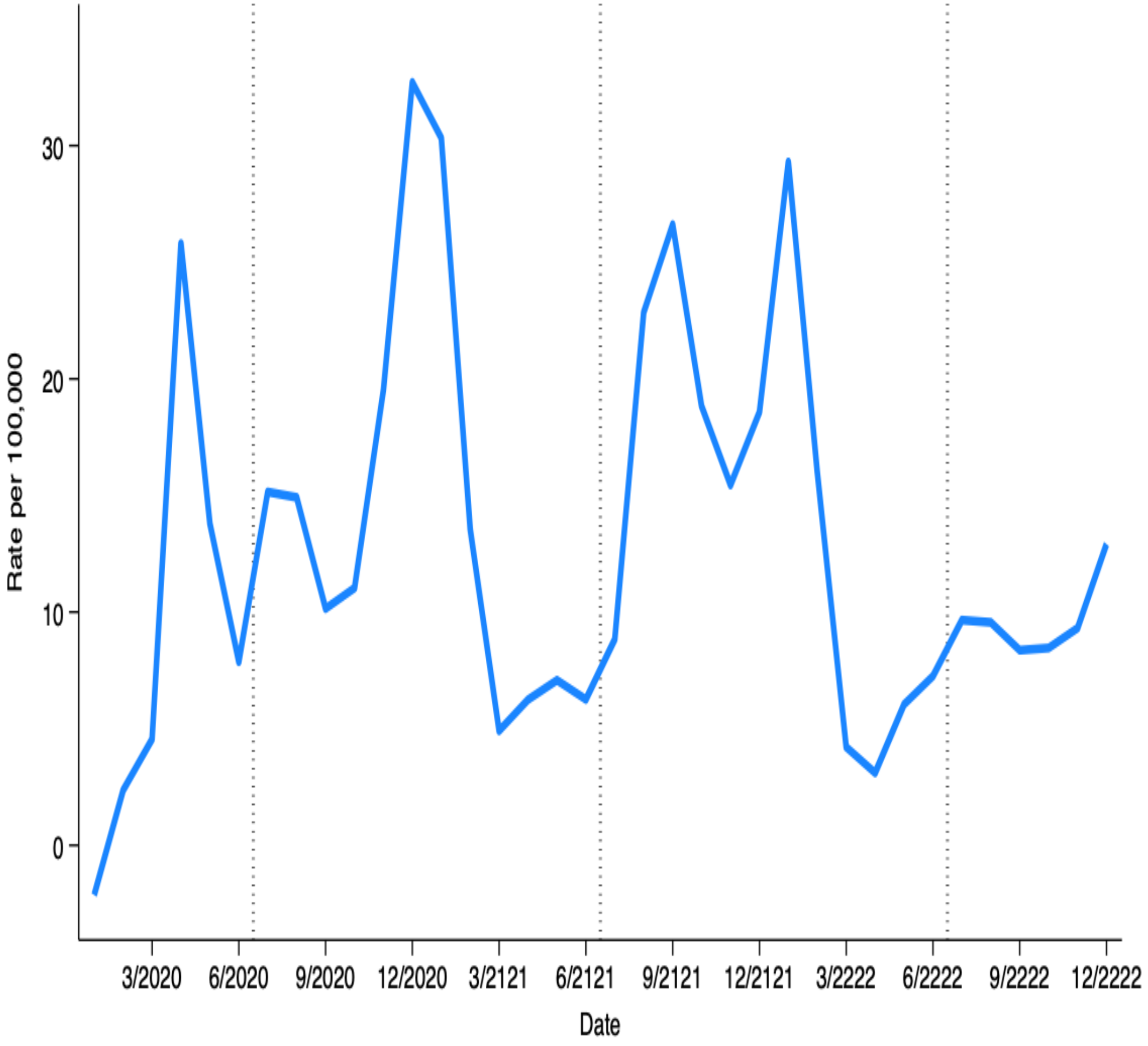

Note: eFigure shows monthly excess death rates per 100,000, computed as age-standardized excess death rates minus corresponding average mortality rates over the 2017-2019 period. Dotted vertical lines show analysis Years 1 (7/20-6/21) and 2 (7/21-6/22).

eFigure 2. Monthly Excess Death Rates and Ratios for States with Below and Above Median Restriction Scores, With and Without CT, MA, NJ, and NY

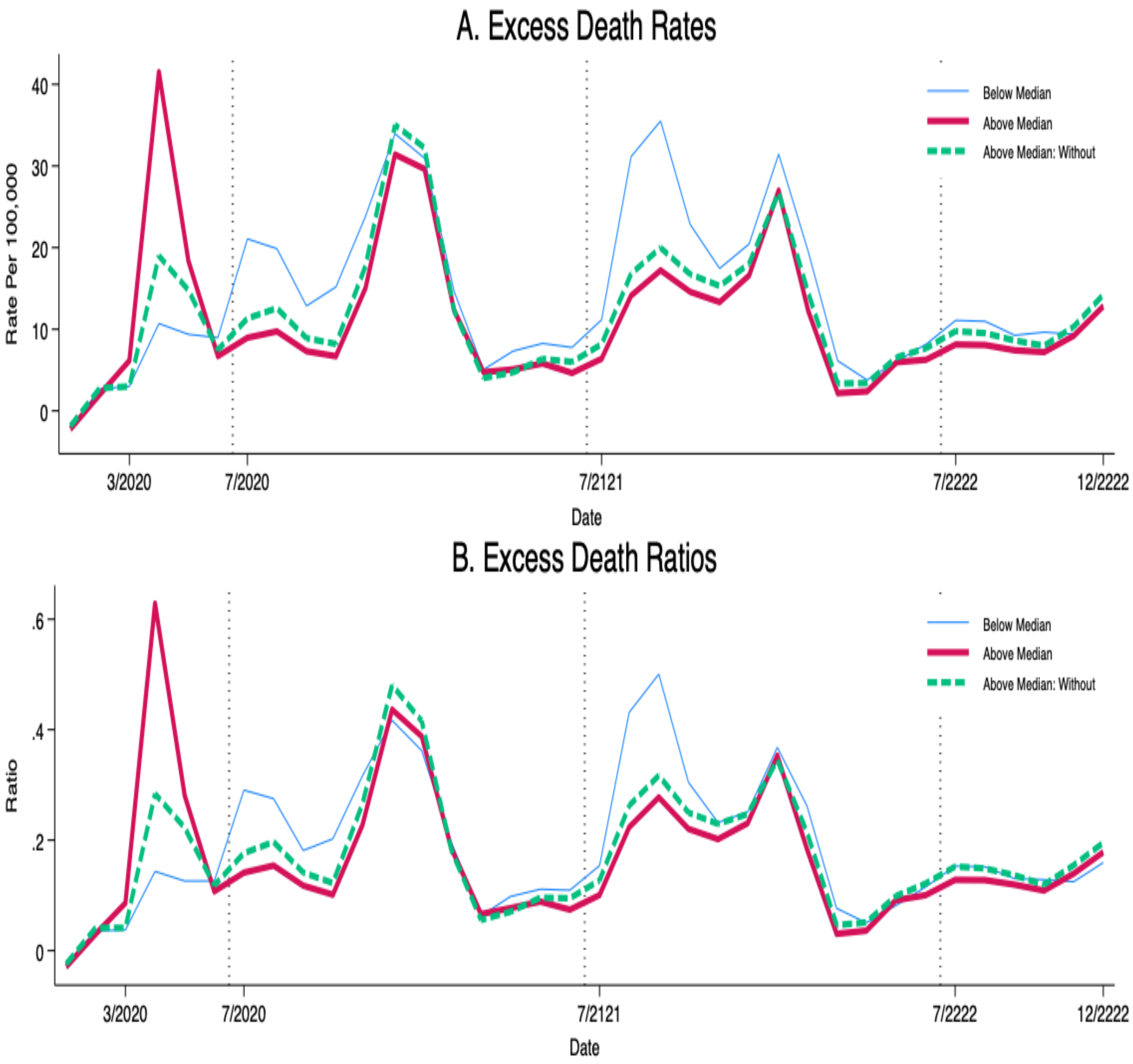

Note: eFigure shows age-standardized monthly excess death rates and ratios for states with below and above median restriction scores, calculated as the sum of population-weighted average values of standardized activity limitations scores, standardized mask requirements, mask and vaccination mandates, minus state prohibitions on vaccination mandates and mask mandates in schools. However, the “Above Median: Without” group excludes CT, MA, NJ, and NY. Dotted vertical lines show analysis years 1 (7/20-6/21) and 2 (7/21-6/22).

© 2024 Ruhm CJ. *JAMA Health Forum*.

eFigure 3. Comparison of Activity Limitation Score with MultiState and WalletHub Mandate Scores

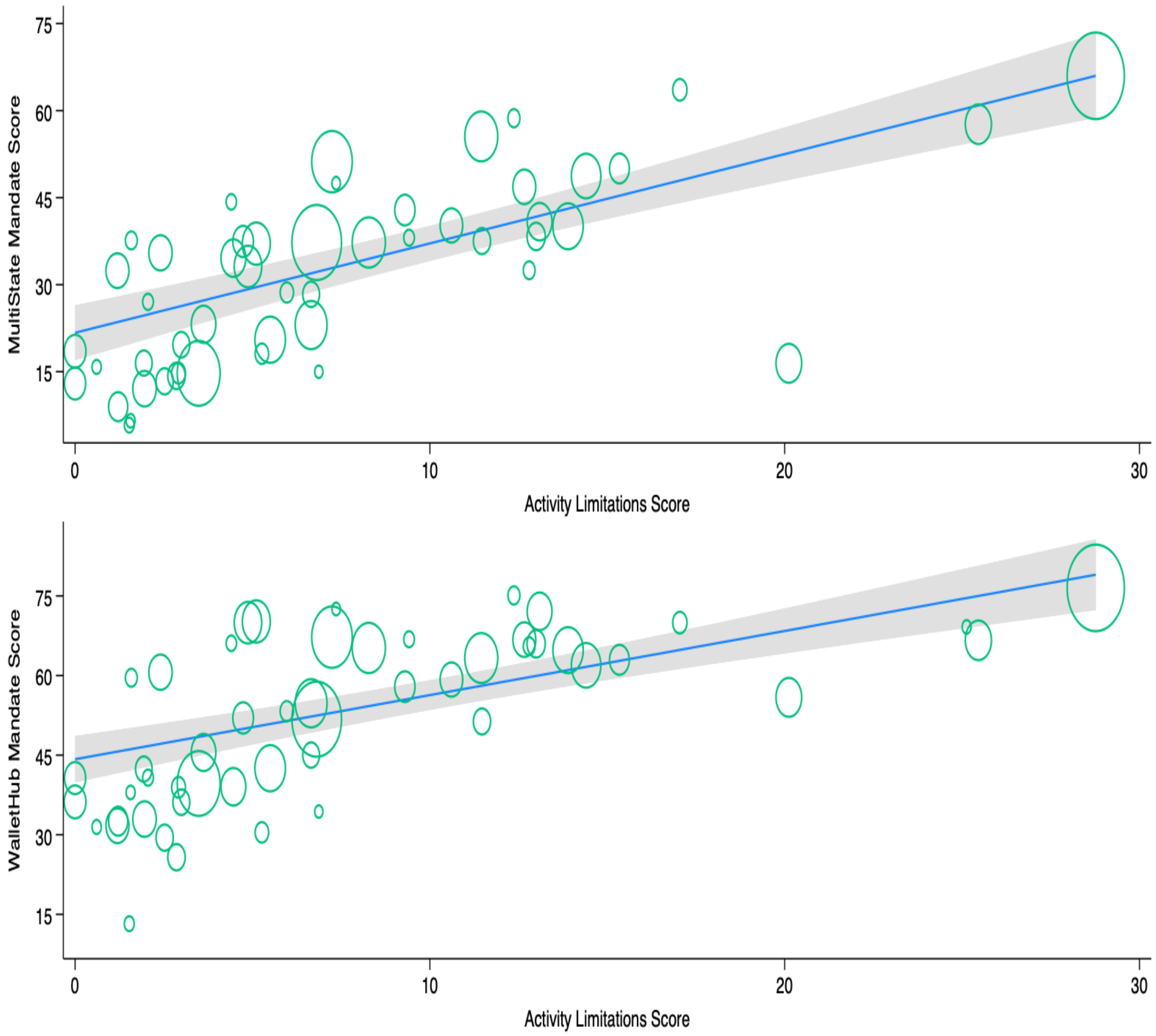

Note: eFigure shows state-level relationship between the activity limitation score measure used in this analysis and corresponding restrictions measured using the MultiState (top panel) and WalletHub (lower panel) data. Circles are proportional to state population size. The line and shadings show estimated relationship and 95% confidence intervals from regressing the restriction score on either the MultiState or WalletHub score. The population-weighted correlation restriction the IHME and Multistate (WalletHub) score is 0.783 (0.728).

eTable 1. Sample Means for States with Strong and Weak Restrictions

Variable	All States	Weak	Strong	Difference
Excess Death Rate (per 100,000)	355.2	403.5	285.6	-117.9
Excess Death Ratio	0.206	0.226	0.188	-0.038
Restrictions				
Activity Limitations: Raw	0.102	0.034	0.194	0.160
Activity Limitations: Standardized	0.000	-0.796	1.069	1.864
Masks Required: Raw	0.437	0.023	0.808	0.786
Masks Required: Standardized	0.000	-1.411	1.264	2.675
State Vaccination Mandate	0.513	0.000	1.000	1.000
School Vaccination Mandate	0.309	0.000	1.000	1.000
School Mask Mandate	0.366	0.000	1.000	1.000
State Vaccination Mandate Prohibited	0.308	0.791	0.000	-0.791
School Mask Mandate Prohibited	0.221	0.748	0.000	-0.748
Behaviors				
Mask Use	0.493	0.432	0.579	0.147
Mobility Reduction	0.075	0.063	0.091	0.028
Fully Vaccinated	0.361	0.332	0.397	0.065

Note: eTable shows population-weighted mean values for all states and for the 10 states with the strongest and weakest COVID-19 restriction scores, calculated as the sum of population-weighted average values of standardized activity limitations scores, standardized mask requirements, mask and vaccination mandates, minus state prohibitions on vaccination mandates and mask mandates in schools. Excess death rates per 100,000 ratios are age-standardized and are for 7/20-6/22. Ratios refer to corresponding ratios of age-standardized deaths to pre-pandemic means. Activity limitations score indicates the average population-weighted share of individuals subject to seven types of activity restrictions. Mask requirements indicate the share required to wear a mask outside the home. Standardized values of these variables are expressed as standard deviations from the national mean. Vaccination mandates and prohibitions indicate if the state had ever put these in place during the two years. Mask use refers to the daily share of adults stating they always wear a mask when leaving home. Mobility reductions indicate share reductions in time away from home, relative to year before the pandemic. The vaccination share refers to those having received one dose of the Johnson & Johnson vaccine or two doses of the Pfizer-BioNTech or Moderna vaccines. The states classified as having weak restrictions are: FL, GA, ID, IA, OK, SC, SD, TN, TX, and UT. Those with strong restrictions are: CA, CT, DC, DE, HI, IL, NM, NY, OR, and WA. Difference refers to the difference in sample means for states with strong versus weak restrictions.

eTable 2. COVID-19 Deaths and Death Ratios: March 2020 - May 2020, by State

State	# Deaths	Population	% of Deaths	% of Population	Death/Population Ratio
New York	28,787	19,336,776	27.7	5.9	4.73
New Jersey	12,099	8,882,371	11.7	2.7	4.33
Connecticut	3,840	3,557,006	3.7	1.1	3.43
Massachusetts	6,667	6,893,574	6.4	2.1	3.07
District of Columbia	500	712,816	0.5	0.2	2.23
Rhode Island	703	1,057,125	0.7	0.3	2.11
Louisiana	2,595	4,645,318	2.5	1.4	1.77
Michigan	5,106	9,966,555	4.9	3.0	1.63
Delaware	466	986,809	0.4	0.3	1.50
Pennsylvania	5,905	12,783,254	5.7	3.9	1.47
Maryland	2,661	6,055,802	2.6	1.8	1.40
Illinois	5,327	12,587,530	5.1	3.8	1.34
Indiana	2,027	6,754,953	2.0	2.1	0.95
Mississippi	720	2,966,786	0.7	0.9	0.77
Colorado	1,307	5,807,719	1.3	1.8	0.71
New Hampshire	295	1,366,275	0.3	0.4	0.69
Minnesota	1,053	5,657,342	1.0	1.7	0.59
Virginia	1,574	8,590,563	1.5	2.6	0.58
Ohio	2,133	11,693,217	2.1	3.5	0.58
Georgia	1,931	10,710,017	1.9	3.3	0.57
New Mexico	372	2,106,319	0.4	0.6	0.56
Iowa	533	3,163,561	0.5	1.0	0.54
Alabama	774	4,921,532	0.7	1.5	0.50
Washington	1,027	7,693,612	1.0	2.3	0.42
Missouri	814	6,151,548	0.8	1.9	0.42
Arizona	928	7,421,401	0.9	2.3	0.40
Nevada	387	3,138,259	0.4	1.0	0.39
California	4,349	39,368,078	4.2	11.9	0.35
Nebraska	214	1,937,552	0.2	0.6	0.35
Florida	2,367	21,733,312	2.3	6.6	0.35
Kentucky	474	4,477,251	0.5	1.4	0.34
South Carolina	547	5,218,040	0.5	1.6	0.33
Wisconsin	607	5,832,655	0.6	1.8	0.33
Vermont	63	623,347	0.1	0.2	0.32
North Carolina	931	10,600,823	0.9	3.2	0.28

North Dakota	63	765,309	0.1	0.2	0.26
South Dakota	70	892,717	0.1	0.3	0.25
Oklahoma	309	3,980,783	0.3	1.2	0.25
Kansas	225	2,913,805	0.2	0.9	0.25
Maine	104	1,350,141	0.1	0.4	0.24
Texas	1,876	29,360,759	1.8	8.9	0.20
Tennessee	386	6,886,834	0.4	2.1	0.18
Idaho	82	1,826,913	0.1	0.6	0.14
Arkansas	134	3,030,522	0.1	0.9	0.14
West Virginia	77	1,784,787	0.1	0.5	0.14
Oregon	177	4,241,507	0.2	1.3	0.13
Utah	113	3,249,879	0.1	1.0	0.11
Wyoming	14	582,328	0.0	0.2	0.08
Montana	20	1,080,577	0.0	0.3	0.06
Alaska	10	731,158	0.0	0.2	0.04
Hawaii	17	1,407,006	0.0	0.4	0.04

Note: eTable shows deaths with COVID-19 as the underlying cause. Ratio indicates the percentage of national COVID-19 deaths occurring in the state divided by the state's percentage of the national 2020 population.

eTable 3. COVID-19 Restrictions and Behaviors by State

State	Activity Limitation Score	Masks Required	State Employee Vaccination Mandate	School Employee Vaccination Mandate	School Mask Mandate	Vaccination Mandate Prohibited	School Mask Mandate Prohibited	Mask Use	Mobility Reduction	Fully Vaccinated
Alabama	0.0	36.7	0	0	0	1	0	42.4	5.7	27.5
Alaska	1.6	0.0	0	0	0	0	0	42.1	5.6	36.1
Arizona	20.1	0.0	0	0	0	1	0	48.8	8.1	34.8
Arkansas	3.0	34.8	0	0	0	1	0	42.7	5.2	29.6
California	28.8	83.4	1	1	1	0	0	60.2	10.1	38.8
Colorado	12.7	41.4	1	0	0	0	0	44.7	7.8	39.0
Connecticut	13.0	83.3	1	1	1	0	0	55.6	7.6	43.9
Delaware	4.4	48.8	1	1	1	0	0	52.6	7.4	38.0
DC	25.1	69.2	1	1	1	0	0	56.6	14.1	44.7
Florida	3.5	0.0	0	0	0	1	1	47.5	7.1	36.5
Georgia	5.5	0.0	0	0	0	1	0	45.2	7.0	29.2
Hawaii	12.4	86.8	1	1	1	0	0	66.1	11.0	43.0
Idaho	5.3	0.0	0	0	0	1	0	31.9	3.3	29.9
Illinois	11.4	72.5	1	1	1	0	0	54.5	7.4	37.7
Indiana	3.6	34.8	0	0	0	0	0	41.5	4.9	31.3
Iowa	2.5	11.4	0	0	0	0	1	35.0	4.2	34.9
Kansas	1.9	37.4	0	0	0	0	0	38.8	4.8	34.6
Kentucky	9.3	46.2	0	0	0	0	0	44.6	5.6	31.8
Louisiana	4.7	51.5	1	0	1	0	0	44.5	5.6	29.3
Maine	12.8	44.9	1	0	0	0	0	46.8	5.1	44.6
Maryland	2.4	43.8	1	0	1	0	0	58.3	10.1	41.5
Massachusetts	13.1	45.6	1	0	1	0	0	53.4	9.7	43.4
Michigan	14.4	48.9	0	0	0	1	0	45.7	6.7	33.8
Minnesota	10.6	40.3	1	0	0	0	0	42.6	8.0	38.8
Mississippi	6.7	7.8	0	0	0	0	0	43.4	5.1	28.2
Missouri	2.0	0.0	0	0	0	0	0	39.6	5.0	31.0
Montana	2.1	29.2	0	0	0	1	0	33.9	3.0	32.4

Nebraska	2.9	0.0	0	0	0	0	0	37.4	5.0	35.7
Nevada	11.5	80.8	1	0	0	0	0	55.4	7.6	33.1
New Hampshire	1.6	20.3	0	0	0	1	0	46.3	7.1	38.9
New Jersey	5.1	45.5	1	1	1	0	0	54.9	9.5	41.4
New Mexico	17.0	81.8	1	1	1	0	0	58.3	7.8	39.9
New York	7.2	80.8	1	1	1	0	0	56.8	8.8	41.6
North Carolina	13.9	43.6	1	0	0	0	0	49.3	6.3	33.4
North Dakota	0.6	9.0	0	0	0	0	0	29.5	3.8	31.4
Ohio	6.7	43.2	0	0	0	0	0	42.3	5.8	32.8
Oklahoma	1.2	0.0	0	0	0	0	1	36.7	5.6	31.6
Oregon	15.3	79.0	1	1	1	0	0	54.2	7.5	38.9
Pennsylvania	8.3	49.7	1	0	0	0	0	45.7	7.4	38.0
Rhode Island	9.4	58.2	1	0	1	0	0	51.3	7.2	45.0
South Carolina	0.0	0.0	0	0	0	0	1	43.5	5.4	30.9
South Dakota	1.5	0.0	0	0	0	1	0	27.4	3.4	34.5
Tennessee	4.5	0.0	0	0	0	1	1	40.5	5.5	29.9
Texas	6.8	34.7	0	0	0	1	1	49.6	8.0	32.8
Utah	2.9	21.0	0	0	0	1	1	38.5	6.2	35.0
Vermont	7.4	43.6	1	0	0	0	0	52.0	7.5	45.9
Virginia	4.9	43.7	1	0	0	0	0	50.9	8.7	39.9
Washington	25.5	84.9	1	1	1	0	0	57.0	8.7	40.1
West Virginia	6.0	47.8	0	0	0	0	0	44.0	4.8	33.1
Wisconsin	1.2	33.3	0	0	0	0	0	41.4	5.8	36.8
Wyoming	6.9	13.4	0	0	0	0	0	26.5	3.0	28.7

Note: Data sources are described elsewhere.

eTable 4. Age-standardized Excess and COVID-19 Death Rates and Ratios

State	Excess Deaths				COVID-19 Deaths			
	Rate	Rank	Ratio	Rank	Rate	Rank	Ratio	Rank
Massachusetts	115.0	1	0.073	1	123.9	4	0.079	5
Hawaii	119.6	2	0.087	3	81.5	2	0.059	2
New Jersey	135.8	3	0.086	2	164.3	10	0.104	11
New York	161.3	4	0.108	6	166.4	11	0.112	16
New Hampshire	173.4	5	0.105	4	123.1	3	0.075	4
Rhode Island	176.2	6	0.106	5	176.3	15	0.106	14
Vermont	186.2	7	0.113	7	64.6	1	0.039	1
Connecticut	202.7	8	0.133	8	145.4	8	0.095	9
Maryland	227.5	9	0.136	9	168.0	12	0.101	10
Minnesota	258.9	10	0.169	13	169.4	13	0.110	15
Maine	262.3	11	0.147	10	130.7	6	0.073	3
Washington	268.4	12	0.170	14	130.1	5	0.082	6
Nebraska	276.1	13	0.162	11	200.9	20	0.118	19
Utah	282.6	14	0.175	17	171.6	14	0.106	13
Illinois	288.9	15	0.170	15	204.6	22	0.121	20
Delaware	292.0	16	0.167	12	184.6	16	0.106	12
North Dakota	297.4	17	0.180	18	253.5	31	0.154	41
Pennsylvania	306.9	18	0.170	16	234.3	27	0.130	26
Idaho	309.9	19	0.181	19	238.2	29	0.139	31
Wisconsin	311.3	20	0.183	20	191.8	18	0.113	17
Iowa	334.4	21	0.193	21	229.3	26	0.133	27
Colorado	337.2	22	0.223	33	188.2	17	0.124	22
California	342.1	23	0.237	39	221.7	23	0.154	40
Virginia	344.8	24	0.206	25	195.3	19	0.117	18
Oregon	347.7	25	0.212	28	140.0	7	0.085	7
Florida	356.5	26	0.225	36	225.1	25	0.142	32
Michigan	359.0	27	0.196	22	224.4	24	0.123	21
DC	374.9	28	0.223	34	158.6	9	0.094	8
South Dakota	376.3	29	0.222	30	264.0	33	0.156	43
Kansas	379.3	30	0.207	26	277.3	36	0.152	38
Montana	382.9	31	0.223	32	249.0	30	0.145	34
Indiana	391.1	32	0.199	23	271.3	35	0.138	30
Ohio	404.6	33	0.205	24	291.5	39	0.148	36
North Carolina	405.2	34	0.223	31	235.6	28	0.129	25
Missouri	407.4	35	0.210	27	266.2	34	0.137	29

© 2024 Ruhm CJ. *JAMA Health Forum*.

Alaska	419.7	36	0.261	46	201.5	21	0.125	23
Georgia	447.6	37	0.243	41	280.0	37	0.152	39
Nevada	454.1	38	0.258	45	319.4	46	0.182	48
Louisiana	465.0	39	0.226	37	258.5	32	0.126	24
Wyoming	472.1	40	0.275	48	282.8	38	0.165	46
Alabama	473.6	41	0.219	29	338.2	48	0.156	44
Kentucky	483.3	42	0.223	35	308.4	43	0.142	33
South Carolina	484.0	43	0.252	43	296.6	40	0.154	42
Texas	484.3	44	0.282	49	341.1	49	0.199	51
Arkansas	495.4	45	0.235	38	309.2	44	0.147	35
Tennessee	508.7	46	0.242	40	317.9	45	0.151	37
Arizona	529.9	47	0.335	51	301.6	41	0.191	50
Oklahoma	537.8	48	0.253	44	376.1	51	0.177	47
West Virginia	556.2	49	0.247	42	306.6	42	0.136	28
New Mexico	556.8	50	0.320	50	321.7	47	0.185	49
Mississippi	589.6	51	0.264	47	352.7	50	0.158	45
US	355.2		0.206		236.7		0.138	

Note. eTable shows age-standardized death excess death rates per 100,000, from 7/20-6/22, and as a ratio of average overall age-standardized death rates in the state from 2017-2019. Also shown are corresponding death rates and ratios where COVID-19 is the underlying cause. Age standardized rates and ratios account for differences in the population shares of <45, 45-64, 65-84 and >85 year olds. The final row in the eTable shows national average for the relevant rate or ratio. Table is sorted from lowest-to-highest excess death rates over the two year period.

eTable 5. Estimated Effect of Single COVID-19 Restrictions on Age-Standardized Excess Death Rates, Ratios and Behaviors

Restriction	Excess Death Rate	Excess Death Ratio	Behaviors (Effect Sizes)			Behaviors (Magnitudes)		
			Mask Use	Mobility Reduction	Vaccination	Mask Use	Mobility Reduction	Vaccination
	(1))	(2)	(3)	(4)	(5)	(6)	(7)	(8)
Activity Limitations	-19.4	0.007	0.676***	0.628***	0.385***	4.9***	1.1***	1.6***
	(11.7)	(0.007)	(0.070)	(0.083)	(0.102)	(0.5)	(0.1)	(0.4)
Masks Required	-45.0***	-0.013	0.763***	0.605***	0.597***	5.5***	1.1***	2.5***
	(13.8)	(0.010)	(0.132)	(0.148)	(0.128)	(1.0)	(0.3)	(0.5)
State Vaccination Mandate	-122.4***	-0.042*	1.399***	1.272***	1.478***	10.1***	2.2***	6.3***
	(27.4)	(0.021)	(0.297)	(0.324)	(0.215)	(2.1)	(0.6)	(0.9)
School Vaccination Mandate	-98.6***	-0.019	1.667***	1.320***	1.282***	12.1***	2.3***	5.5***
	(33.5)	(0.026)	(0.201)	(0.307)	(0.224)	(1.5)	(0.5)	(1.0)
School Mask Mandate	-119.0***	-0.035	1.650***	1.380***	1.372***	11.9***	2.4***	5.8***
	(35.4)	(0.029)	(0.218)	(0.294)	(0.227)	(1.6)	(0.5)	(1.0)
State Vaccination Mandate Prohibited	102.4***	0.052**	-0.656*	-0.416	-1.042***	-4.7*	-0.7	-4.4***
	(34.0)	(0.020)	(0.329)	(0.348)	(0.285)	(2.4)	(0.6)	(1.2)
School Mask Mandate Prohibited	92.9**	0.048**	-0.607	-0.380	-0.741**	-4.4	-0.7	-3.2**
	(42.4)	(0.022)	(0.364)	(0.382)	(0.307)	(2.6)	(0.7)	(1.3)

Note: eTable shows coefficient estimates from regressing age-standardized state excess death rates or ratios from 7/20-6/-6/22 or behavioral responses on the specified restriction. In columns (1) and (2), age-specific excess death rates or ratios from 3/20-6/20 are also controlled for. The dependent variables in columns (3) through (5) are standardized behaviors (i.e. transformed to have a standard deviation equal to one), while those in columns (6) through (8) are raw values. Observations are weighted by 2020 state populations and robust standard errors are shown in parentheses. Each cell shows the results of a separate regression. Continuous restrictions are standardized, so that the coefficients indicate the estimated effect of a one standard deviation change. For dummy variables, the coefficient indicates the estimated effect of its value going from 0 to 1.
*p<0.10, **, p<0.05, ***p<0.01.

eTable 6. Estimated Effect of Specific Activity Limitations on Age-Standardized Excess Death Rates and Ratios

Specific Limitation	Excess Death Rate	Excess Death Ratio
Restaurant Closures	-23.8**	0.003
	(9.9)	(0.006)
Bar Closures	-10.7	0.010
	(14.0)	(0.007)
Primary Education Closures	-7.2	0.009
	(19.9)	(0.011)
Higher Education Closures	-26.2	-0.009
	(22.0)	(0.014)
Public Gatherings	-11.4	0.005
	(13.4)	(0.007)
Leisure Activities	-22.0**	0.005
	(10.0)	(0.006)
Stay-at-Home Orders	-15.9**	0.008**
	(6.3)	(0.003)

Note: eTable shows coefficient estimates from regressing age-standardized state excess death rate or ratio from 7/20-6/22 on the specified activity limitation, as well as excess death rates or ratios from 3/30-6/20. Observations are weighted by 2020 state populations and robust standard errors are shown in parentheses. Each cell shows the results of a separate regression. The activity limitations are standardized, so that the coefficients indicate the estimated effect of a one standard deviation change. *p<0.10, **, p<0.05, ***p<0.01.

eTable 7. Estimated Effect of Single Behaviors on Age-Standardized Excess Death Rates or Ratios

Behavior	Excess Death Rate	Excess Death Ratio
Mask Use	-37.0***	-0.000
	(11.3)	(0.007)
Mobility Reduction	-40.0***	0.001
	(12.0)	(0.007)
Fully Vaccinated	-86.8***	-0.029***
	(9.9)	(0.009)

Note: eTable shows coefficient estimates from regressing age-standardized excess death rates or ratios for 7/20-6/22 on the specified behavior, with age-specific excess death rates or ratios from 3/20-6/20 are also controlled for. Observations are weighted by 2020 state populations and robust standard errors are shown in parentheses. Each cell shows the results of a separate regression. The behavior variables are standardized, so that the coefficients indicate the estimated effect of a one standard deviation change.
*p<0.10, **, p<0.05, ***p<0.01.

eTable 8. Robustness Checks for Strong vs. Weak Restriction State Differences in Age-Standardized Excess Death Rates and Ratios

Outcome	(1)	(2)	(3)	(4)	**(5)**	(6)	(7)	(8)
Excess Death Rate	-134.8***	-138.9***	-108.8***	-136.8***	-105.0***	-84.5***	-54.7**	-80.1***
	(32.7)	(33.1)	(35.6)	(32.4)	(38.7)	(23.9)	(25.5)	(16.1)
Excess Death Ratio	-0.048***	-0.048***	-0.024	-0.051***	-0.030**	-0.031**	-0.030	-0.065***
	(0.016)	(0.014)	(0.026)	(0.013)	(0.015)	(0.013)	(0.030)	(0.016)
Specification Details	Main	Specific Activity Limits	Overall Restriction Score	No “Early” Controls	Full Pandemic	COVID-19 Deaths	Year 1	Year 2

Note: eTable shows estimated differences in age-standardized excess or COVID-19 death rates and ratios. The analysis period is 7/20-6/22, except in columns 5, 7, and 8. Weak restrictions refer to population-weighted average values of the restrictions included for the 10 states with the lowest overall restriction scores. Strong restrictions refer to corresponding population-weighted averages for the 10 states with the highest overall restriction scores. Column (1) repeats the main specification from Table 3. In column (2), the seven specific activity limitations are controlled for, rather than just the overall average. Column (3) controls only for the single overall restriction score (described in Table 2). Column (4) shows results where excess death rates or ratios from 3/20-6/20 are not included in the set of controls. Column (5) shows results for the full pandemic period (3/20-6/22) but with CT, MA, NJ, and NY and controls for excess death rates or ratios for 3/20-6/20 excluded. The dependent variable in column (6) refers to deaths with COVID-19 as the underlying cause. The analysis periods in columns (7) and (8) are 7/20-6/21 and 7/21-6/22, respectively. Robust standard errors are shown in parentheses.
*p<0.10, **, p<0.05, ***p<0.01

eTable 9. Estimated Age-Standardized Excess Death Rates and Ratios for States with Weak and Strong COVID-19 Restrictions

	Excess Death Rate			Excess Death Ratio		
	Weak	Strong	Difference	Weak	Strong	Difference
Overall	417.0***<br>(28.1)	282.2***<br>(15.8)	-134.8***<br>(32.7)	0.236***<br>(0.012)	0.188***<br>(0.009)	-0.048***<br>(0.016)
Through Behaviors	403.1***<br>(13.0)	297.3***<br>(11.1)	-105.9***<br>(17.6)	0.219***<br>(0.009)	0.196***<br>(0.007)	-0.023**<br>(0.012)

Note: “Overall” shows estimated age-standardized excess death rates and ratios for 7/20-6/22 for states with weak and strong COVID-19 restrictions. “Through Behaviors” shows corresponding estimated rates and ratios from 7/20-6/22 due to behaviors associated with weak and strong COVID-19 restrictions, and the difference between them. Weak restrictions refer to population-weighted average values of standardized activity limitations scores, standardized mask requirements, mask and vaccination mandates, and state prohibitions on vaccination mandates and mask mandates in schools for the 10 states with the lowest overall restriction scores. Strong restrictions refer to corresponding population-weighted averages for the 10 states with the highest overall restriction scores. Overall effects are estimated from population-weighted regressions of the outcomes on these variables plus age-specific excess death rates or ratios from 3/20-6/20. “Through Behaviors” are estimated from population-weighted regressions of behaviors on restrictions and of excess death rates and ratios on the behaviors and excess death rates or ratios from 3/20-6/20. For calculating marginal effects, estimated values of behaviors from the first set of regressions are used in the second-stage. Robust standard errors are shown in parentheses.

*p<0.10, **, p<0.05, ***p<0.01

eTable 10. Estimated Behaviors in States with Weak and Strong COVID-19 Restrictions

	Weak	Strong	Difference
Effect Sizes			
Mask Use	6.09***	8.02***	1.94***
	(0.15)	(0.06)	(0.17)
Mobility Reductions	3.79***	5.26***	1.47***
	(0.15)	(0.16)	(0.20)
Vaccinations	7.85***	9.34***	1.50***
	(0.26)	(0.13)	(0.30)
Effect Magnitudes			
Mask Use	44.0***	58.0***	14.0***
	(1.1)	(0.4)	(1.2)
Mobility Reductions	6.6***	9.2***	2.6***
	(0.3)	(0.3)	(0.4)
Vaccinations	33.4***	39.8***	6.4***
	(1.1)	(0.5)	(1.3)

Note: eTable shows estimated behaviors, from 7/20-6/22, for states with weak and strong COVID-19 restrictions. Weak restrictions refer to population-weighted average values of standardized activity limitations scores, standardized mask requirements, mask and vaccination mandates, and state prohibitions on vaccination mandates and mask mandates in schools for the 10 states with the lowest overall restriction scores. Strong restrictions refer to corresponding population-weighted averages for the 10 states with the highest overall restriction scores. These the ”first-stage” for the estimates in Figure 4 and eTable 5 and are estimated from population-weighted regressions of the outcomes on these variables. Difference indicates estimated difference between strong versus weak restrictions; the p-value refers to this difference. Robust standard errors are shown in parentheses. The top panel shows estimated effects in terms of standard deviation changes; the bottom shows them in terms of absolute changes.

*p<0.10, **, p<0.05, ***p<0.01

eTable 11. State to US Mortality Rate Ratios and Selected State Characteristics

State	Mortality Rate Ratio	Bachelor's Degree	Medicaid Expansion	TANF Benefits
Hawaii	0.801	0	Yes	$610
California	0.843	0	Yes	$879
New York	0.869	0	Yes	$789
Colorado	0.884	0	Yes	$508
Connecticut	0.892	0	Yes	$597
Minnesota	0.898	0	Yes	$532
Massachusetts	0.918	0	Yes	$633
Washington	0.922	0	Yes	$569
New Jersey	0.922	0	Yes	$559
Florida	0.925	0	No	$303
Arizona	0.925	0	Yes	$278
Alaska	0.940	0	Yes	$923
Utah	0.945	0	Yes	$498
Oregon	0.958	0	Yes	$506
Vermont	0.960	0	Yes	$640
North Dakota	0.964	0	Yes	$486
New Hampshire	0.965	0	Yes	$1,066
Rhode Island	0.970	0	Yes	$554
Maryland	0.977	0	Yes	$709
Virginia	0.980	0	Yes	$442
District of Columbia	0.983	1	Yes	$642
South Dakota	0.990	0	No	$615
Illinois	0.991	0	Yes	$520
Wisconsin	0.993	0	No	$608
Nebraska	0.994	0	Yes	$468
Idaho	0.999	0	Yes	$309
Texas	1.004	0	No	$295
Montana	1.004	0	Yes	$588
Wyoming	1.004	0	No	$697
Iowa	1.011	0	Yes	$426
New Mexico	1.017	0	Yes	$447
Delaware	1.021	0	Yes	$338
Nevada	1.028	0	Yes	$386
Maine	1.044	0	Yes	$594
Pennsylvania	1.053	0	Yes	$403
North Carolina	1.064	0	No	$272
Michigan	1.069	0	Yes	$492
Kansas	1.070	0	No	$429
Georgia	1.077	0	No	$280

South Carolina	1.124	0	No	$292
Missouri	1.132	0	Yes	$292
Indiana	1.148	0	Yes	$288
Ohio	1.154	0	Yes	$497
Louisiana	1.203	0	Yes	$240
Tennessee	1.227	0	No	$277
Arkansas	1.231	0	Yes	$204
Oklahoma	1.243	0	No	$292
Alabama	1.264	0	No	$215
Kentucky	1.267	0	Yes	$262
Mississippi	1.308	0	No	$170
West Virginia	1.316	0	Yes	$374

Note: Mortality rate ratio is the ratio of state-to-US all-cause age-standardized mortality rates from 2017-2019; table is sorted from smallest-to-largest ratio. Bachelor's Degree indicates the share of state residents ≥25 with at least a Bachelor's degree in 2000. Medicaid expansion indicates whether the state had expanded Medicaid under the Affordable Care Act by January 1, 2020. TANF benefits indicate the state benefit in 2019 for a single parent family with two children.

eTable 12. Components of Alternative Activity Limitations Measures

<table>
<tr><th>Type of Limitation</th><th>IHME</th><th>Multi-State</th><th>Wallet-Hub</th></tr>
<tr><td>Non-essential office restrictions</td><td></td><td>✓</td><td></td></tr>
<tr><td>Non-essential retail services restrictions</td><td></td><td>✓</td><td rowspan="2">✓</td></tr>
<tr><td>Personal care services restrictions</td><td></td><td>✓</td></tr>
<tr><td>Personal fitness/leisure activity restrictions</td><td>✓</td><td>✓</td><td></td></tr>
<tr><td>Restaurants restrictions</td><td>✓</td><td>✓</td><td rowspan="2">✓</td></tr>
<tr><td>Bar restrictions</td><td>✓</td><td>✓</td></tr>
<tr><td>Venues serving large crowd restrictions</td><td></td><td>✓</td><td></td></tr>
<tr><td>Construction site restrictions</td><td></td><td>✓[3]</td><td></td></tr>
<tr><td>Stay-at-home/Shelter-in-place orders</td><td>✓</td><td>✓</td><td>✓</td></tr>
<tr><td>State presumption of restrictive local measures</td><td></td><td>✓</td><td></td></tr>
<tr><td>Definition of essential businesses</td><td></td><td>✓[3]</td><td></td></tr>
<tr><td>Mandatory curfews</td><td></td><td>✓[4]</td><td></td></tr>
<tr><td>Public/private gatherings</td><td>✓</td><td>✓[4]</td><td>✓</td></tr>
<tr><td>Business immunity from COVID-19 claims</td><td></td><td></td><td>✓</td></tr>
<tr><td>Public mask requirements</td><td>✓[1]</td><td></td><td>✓</td></tr>
<tr><td>Court closures</td><td></td><td></td><td>✓</td></tr>
<tr><td>School closures</td><td>✓[2]</td><td></td><td>✓</td></tr>
<tr><td>Work-from-home requirements</td><td></td><td></td><td>✓</td></tr>
<tr><td>Mandated contact tracing</td><td></td><td></td><td>✓</td></tr>
<tr><td>Multi-state reopening agreements</td><td></td><td></td><td>✓</td></tr>
<tr><td>Consumer health checks at restaurants</td><td></td><td></td><td>✓</td></tr>
<tr><td>Travel restrictions</td><td></td><td></td><td>✓</td></tr>
<tr><td>Elective medical procedures</td><td></td><td></td><td>✓</td></tr>
<tr><td>Assisted living facilities</td><td></td><td></td><td>✓</td></tr>
<tr><td>Workplace temperature screenings</td><td></td><td></td><td>✓</td></tr>
<tr><td>Penalties for COVID-19 legislation noncompliance</td><td></td><td></td><td>✓</td></tr>
</table>

[1] Mask mandates from other sources are used in the main analysis
[2] Separate restrictions for primary and higher education facilities
[3] In effect before 2/8/21
[4] In effect starting 2/8/21
