# Supplementary material for: US State Restrictions and Excess COVID-19 Pandemic Deaths
Source: JAMA Health Forum. 2024 Jul 26;5(7):e242006. doi: 10.1001/jamahealthforum.2024.2006 (PMC11282449; doi:10.1001/jamahealthforum.2024.2006)
Supplement: Supplement 2. — Data Sharing Statement [file jamahealthforum-e242006-s002.pdf]

Data Sharing Statement

Ruhm. US State Restrictions and Excess COVID-19 Pandemic Deaths. *JAMA Health Forum*. Published July 26, 2024. doi:10.1001/jamahealthforum.2024.2006

Data
Data available: Yes
Data types: Data (not involving human participants)
How to access data: Request from ruhm@virginia.edu
When available: With publication

Supporting Documents
Document types: Statistical/analytic code
How to access documents: ruhm@virginia.edu
When available: With publication

Additional Information
Who can access the data: Any one requesting data.
Types of analyses: Any purpose
Mechanisms of data availability: After approval of a proposal
Any additional restrictions: None
